# Longitudinal PET Imaging Reveals the Biological Fate, Pharmacokinetics, and Clearance of Zirconium-89-Labeled MXene Nanosheets *In Vivo*

**DOI:** 10.7150/ntno.138975

**Published:** 2026-10-09

**Authors:** Iqra Bibi, Sairan Eom, Sang Jin Han, Kyung Jun Kang, Jung Young Kim, Sajid Mushtaq, Ji-Ae Park

**Affiliations:** 1 Division of Applied RI, Korea Institute of Radiological & Medical Sciences (KIRAMS), 75 Nowon-ro, Nowon-gu, Seoul 01812, Republic of Korea.; 2 University of Science and Technology (UST), 217, Gajeong-ro, Yuseong-gu, Daejeon 34113, Republic of Korea.; 3 Department of Chemistry, Pakistan Institute of Engineering and Applied Sciences (PIEAS), P.O. Nilore, Islamabad 45650, Pakistan.

**Keywords:** MXene nanosheets, biological fate, zirconium-89 PET imaging, nano-bio interaction, nanotheranostic

## Abstract

Two-dimensional MXene (Ti_3_C_2_Tx) nanosheets have emerged as versatile platforms for drug delivery, photothermal and photodynamic therapy, combined chemo-photothermal treatment, and multimodal imaging including MRI and CT, as well as theranostic applications. However, their clinical translation is limited by an incomplete understanding of their long-term *in vivo* fate, pharmacokinetics, and biological interactions following systemic administration. Here, we developed a stable zirconium-89-labeled MXene platform via DFO-mediated chelation to enable quantitative, noninvasive, and longitudinal positron emission tomography (PET) imaging over 21 days. Comprehensive physicochemical characterization confirmed successful synthesis, surface functionalization, and radiolabeling of MXene nanosheets, yielding a radiochemical purity of 99.39 ± 0.66% and serum stability exceeding 97.3±1.9% over 7 days. PET/CT imaging and biodistribution studies revealed an initial pulmonary accumulation phase (30.43 ± 5.39 %ID/g), followed by progressive sequestration within the reticuloendothelial system, predominantly in the liver (maximum 30.16 ± 5.42 %ID/g on day 7) and spleen (9.31±3.17 %ID/g on day 7). Quantitative biodistribution analysis further demonstrated prolonged organ retention with gradual elimination through both renal and hepatobiliary pathways, as confirmed by excretion studies. Minimal skeletal uptake (<1.04±0.14 %ID/g throughout the study) indicated excellent *in vivo* radiolabel stability. Histopathological examination of major organs, together with *in vitro* cytotoxicity assays, revealed no significant adverse effects despite prolonged tissue retention. Collectively, these findings provide insight into the nano-bio interactions governing MXene biodistribution, organ accumulation, and clearance. More broadly, this study establishes a PET-based framework for evaluating the biological fate and safety of emerging two-dimensional nanomaterials and supports the rational development of MXene-based nanotheranostic platforms for biomedical applications.

## 1. Introduction

Achieving effective and personalized cancer treatment requires the coordinated integration of diagnosis, therapy, and treatment monitoring. Nanotheranostic platforms have emerged as powerful tools for this purpose 1. They can simultaneously deliver therapeutic agents and provide imaging capabilities that enable real-time visualization of biological processes *in vivo* 2. Such multifunctional systems have the potential to improve target specificity, reduce systemic toxicity, and facilitate image-guided therapeutic interventions 3. Despite remarkable advances in nanomaterial engineering, the clinical translation of many nanotheranostic agents remains limited by an incomplete understanding of their *in vivo* fate 4. In particular pharmacokinetics, biodistribution, organ retention, and clearance behavior are key determinants of both therapeutic efficiency and safety. Consequently, quantitative and longitudinal assessment of nanomaterial behavior in living subjects has become a central requirement for the development of clinically translatable nanotheranostic system 5. To achieve this goal, robust imaging technologies capable of quantitative monitoring nanomaterial behavior in living subjects are required.

Among the available imaging modalities, positron emission tomography (PET) is uniquely suited for investigating the pharmacokinetics and distribution of nanomedicine. It combines exceptional sensitivity, unlimited tissue penetration, and quantitative whole-body imaging capabilities 6. Unlike fluorescence-based approaches, which are often affected by tissue attenuation, photobleaching, and limited penetration depth 7, PET enables accurate longitudinal tracking of radiolabeled nanomaterial *in vivo*. Furthermore, hybrid PET/CT imaging provides both functional and anatomical information, allowing precise localization and quantification of nanomaterial accumulation in the individual organs 8. These capabilities have established PET as a powerful tool for evaluating circulation kinetics, target-site accumulation, clearance pathways, and biological interactions of diverse nanomedicine platforms. Particularly for long-circulating nanomaterials, radionuclides with extended physical half-lives, such as Zirconium-89 (T_1/2_ = 78.4h), offer unique opportunities for longitudinal assessment of biological fate over days to weeks 9. This capability provides critical insights that cannot be obtained using conventional imaging modalities such as MRI and CT or *ex vivo* analytical techniques such as inductively coupled plasma mass spectrometry (ICP-MS), respectively 10.

Among emerging two-dimensional materials, MXenes have attracted substantial attention as multifunctional platform for theranostic and biomedical applications 11-12. Since their discovery, titanium carbide (Ti_3_C_2_Tx)-based MXenes have demonstrated remarkable potential for drug delivery 13, molecular imaging 14, biosensing 15, and photodynamic and photothermal therapy 16. These applications are enabled by their ultrathin layered structure, large surface area, excellent photothermal conversion efficiency, and readily modifiable surface chemistry 17. The presence of abundant surface terminal groups (-OH, -O, and -F) facilitate versatile functionalization strategies, enabling the incorporation of therapeutic agents, imaging probes and targeting ligands 18. These characteristics have positioned MXene among the most promising nanomaterials for the development of next generation theranostic system 19. However, despite their rapidly expanding biomedical applications, the biological behavior of MXene nanosheets following systemic administration remains incompletely understood 20. Because the therapeutic efficiency and safety of nanomaterials are intrinsically linked to their pharmacokinetics, biodistribution, organ retention, and clearance characteristics, comprehensive, evaluation of their *in vivo* fate is essential before clinical translation can be realized 21.

Several studies have investigated the biocompatibility of MXene nanosheets, reporting predominant accumulation within organs of the reticuloendothelial system, including the lungs, liver, and spleen 22-24. While these investigations have provided valuable preliminary insights, critical questions regarding long-term organ retention, clearance mechanism, systemic toxicity, and dynamic biological interactions remain largely unresolved. Most existing studies have relied on* in vitro* assays, endpoint histological analyses, fluorescence imaging, or elemental quantification techniques such as ICP-MS 25. Although useful, these approaches are unable to provide continuous, real-time visualization of nanomaterial behavior in living subjects. Fluorescence-based methods suffer from limited tissue penetration, photobleaching, and poor quantitative accuracy 26-28, whereas ICP-MS measurements provide only static endpoint information and cannot capture longitudinal pharmacokinetics processes 29-30. Consequently, the long-term biological fate of MXene nanosheets remains poorly defined, creating a significant barrier to their rational design translation as nanotheranostic agents.

To overcome the current limitations in the MXene biodistribution assessment, we engineered a PET-trackable MXene nanoplatform through stable ^89^Zr radiolabeling (Figure 1) and performed longitudinal PET/CT imaging for up to 21 days following systemic administration. This approach enabled noninvasive and quantitative monitoring of MXene circulation, tissue distribution, organic sequestration, clearance, and long-term retention in living subjects. By integrating physicochemical characterization, radiolabeling validation, biodistribution analysis, excretion studies we comprehensively investigated the biological fate of MXene nanosheets and their interaction with host biological system. This study provides fundamental insight into the pharmacokinetics behavior and safety profile of MXene nanomaterials and establish a translational framework for the future development of MXene-based molecular imaging agents, drug delivery systems, and nanotheranostic platforms.

## 2. Materials and Methods

### 2.1. Reagents and Chemicals

Ti₃AlC₂, LiF (99.9%), HCl (36-38%), DMSO (> 99.9%), ethanol (95%), and APTES (99%) were obtained from Sigma Aldrich (Republic of Korea). The chelating agent *p*-SCN-Bz-DFO was procured from Macrocyclics (USA). Ultrapure water (18.2 MΩ·cm) was supplied by a Thermo purification system. Unless otherwise specified, all chemicals were of analytical grade and used as received without additional purification.

### 2.2. Synthesis of MXene

MXene was synthesized from the MAX phase using a mild etching method with 12M LiF/9M HCl solution, following previously reported procedures 31,32. Briefly, 0.8 g of LiF was dissolved in 10 mL of 9 M HCl and stirred for 5 min. MAX-phase powder (0.5 g) was then slowly added to the solution over a period of 5 minutes. The reaction mixture was stirred at room temperature for 36 h. The resulting suspension was washed with Deionized (DI) water by repeated centrifugation (3500 rpm, 5 min) until the pH reached ≥ 5. The black MXene layer at the bottom was carefully separated from the unetched gray MAX phase residue, which was then vacuum-filtered and dried at 120 °C for 24 h.

### 2.3. Synthesis of MXene-APTES

Following MXene synthesis, amino-functionalization was performed using APTES, a widely employed aminosilane reagent for surface silanization. The delaminated MXene (5 mg mL^-1^, 5 mL) was dispersed in a 55 mL ethanol/water mixture (10 vol% ethanol) and ultrasonicated for 30 min to ensure uniform dispersion. Subsequently, an excess of APTES (2 mL) was slowly added to the suspension, which was then stirred at room temperature for 24 h. The resulting product was collected by centrifugation and thoroughly washed with ethanol and DI water to remove unreacted APTES. Finally, MXene-APTES was obtained as a black powder after freeze-drying, which was stored under an argon atmosphere, and characterized by XRD, FTIR, and XPS analyses 33-35.

### 2.4. Synthesis of MXene-APTES-DFO

Conjugation of* p*-SCN-Bz-DFO to MXene-APTES was performed under mild basic conditions 36,37. Briefly, 2.5 mg of MXene-APTES was dispersed in 0.5 M HEPES buffer (pH 7.4), and the pH of the reaction mixture was adjusted to 8.8-9.0 by adding small aliquots (< 10 µL) of 0.1 M sodium carbonate (Na₂CO₃) solution. A *p*-SCN-Bz-DFO stock solution (5 mM, 3.8 mg/mL) was prepared in DMSO, 20 µL of that stock solution was added to the MXene-APTES suspension in four portions (5 µL each) at 2-3 min intervals under continuous stirring, while maintaining the DMSO amount at ≤ 2% v/v. The reaction mixture was then incubated at 37 °C for 30 min with gentle agitation (550 rpm). Upon completion, the product was washed thoroughly with DI water, followed by ethanol washing to remove unreacted *p*-SCN-Bz-DFO and other impurities. The purified MXene-APTES-DFO conjugate was dried under vacuum and characterized by FTIR and XPS.

### 2.5. Characterization of MXene and Its Variants

The surface functionalization of all samples was characterized using FTIR spectroscopy to confirm the presence of functional groups introduced at each modification step. For FTIR measurements, samples were placed directly onto the crystal of the spectrometer, and the spectra were recorded without any additional sample preparation. Data were acquired using a Nicolet iS5 FTIR spectrometer (Thermo Fisher Scientific), and the resulting spectra were processed and analyzed using Origin software. The surface chemistry of MXene, MXene-APTES, and MXene-APTES-DFO was further analyzed using XPS-UPS (Nexsa). Al Kα radiation (hν = 1486.6 eV, 200 W) served as the excitation source. The C 1s signal at 285.0 eV, corresponding to C-C/C-H bonds, was used as an internal standard for the quantitative analysis of different chemical species. The hydrodynamic size distributions of MXene and MXene-APTES-DFO sheets were measured using Dynamic light scattering (DLS) with an Ozum Nano ZS analyzer (Japan) equipped with a backscattering light detector at 173°. Samples were suspended in DI water. Each sample was homogenized for 30 seconds by ultrasonication before analysis. Measurements were performed at 25 °C temperature, and each measurement was repeated three times; the results are reported as the mean ± standard deviation.

The stabilities of the MXene, MXene-APTES, and MXene-APTES-DFO suspensions in water were evaluated using an Ozum Nano ZS zeta potential analyzer (Japan). Samples were prepared in DI water and homogenized for 30 seconds via ultrasonication before measurement. Measurements were performed at 25 °C, and each measurement was repeated six times; the results are reported as the mean ± standard deviation. The morphologies of the MAX phase expanded MXene, and MXene-APTES-DFO were examined by field-emission scanning electron microscopy (FE-SEM) using a FE-SEM Hitachi Regulus 8230. Homogeneous suspensions in DI water were deposited onto Si wafers and coated with a thin carbon layer before imaging. The structure of MXene-APTES-DFO was further analyzed by TEM using a Cs-corrected scanning transmission electron microscope (STEM) (JEOL NEOARM). Samples were prepared on carbon-coated copper grids and imaged in high-resolution mode. The layered structures were visualized through electron diffraction analysis.

### 2.6. *In Vitro* Cytotoxicity Analysis

The cytotoxicity of MXene-APTES-DFO was evaluated in four cell lines, including, normal lung fibroblasts (MRC-5), human alveolar epithelial cells (A549), human skin fibroblasts (Detroit-551) and human melanoma (A375). MRC-5 and A549 cells were cultured in Minimum Essential Medium (MEM), while Detroit-551 and A375 were maintained in Dulbecco's Modified Eagle Medium (DMEM). Both media were supplemented with 10% fetal bovine serum (FBS), 1% penicillin-streptomycin, and 1% L-glutamine, and cells were incubated at 37 °C in a humidified atmosphere containing 5% CO_2_. Cell viability was assessed using a CCK-8 assay (EZ-Cytox, DoGen, Korea) according to the manufacturer's instructions. Cells were seeded at a density of 1 × 10⁴ per well and allowed to adhere prior to treatment. The culture medium was then replaced with serum-free MEM or DMEM media containing varying concentrations of MXene-APTES-DFO (100 μL per well), followed by incubation for 24 h. Untreated cells served as controls. Following exposure, cells were washed with phosphate-buffered saline (PBS, Sigma-Aldrich), and CCK reagent (10 μL per well) was added. After a 2 h incubation period, absorbance was measured at 450 nm (reference wavelength at 630 nm) using a microplate reader. The samples were prepared in quadruplicates.

Cell viability (%) was calculated using the following equation:

Cell viability (%) =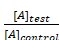
× 100

### 2.7. Synthesis of ^89^Zr-Oxalate

^89^Zr was produced via the ^89^Y (p,n)^ 89^Zr nuclear reaction using a 50 MeV medical cyclotron (Scanditronix, Sweden, 1985) at KIRAMS. Following modified literature protocols 38-39 a solid ^89^Y foil target (20 × 20 × 0.025 mm, ~20 mg) was irradiated with 18-20 MeV proton beam at a current of ≈ 20 μA for 2-3 h. The activated target was dissolved in 6 M HCl (3 mL) and passed through a hydroxamate column for selective isolation of ^89^Zr. Impurities were removed by sequential washing with 2 M HCl and DI water, and purified ^89^Zr was eluted with 1 M oxalic acid (1 mL). The final product, ^89^Zr -oxalate (74-185 MBq/mL), exhibited > 99% radiochemical and radionuclidic purity, confirmed by iTLC and γ-spectrometry using a high-purity germanium detector at 909 keV. The purified ^89^Zr-oxalate was adjusted to 0.1 M (pH 6) using a quaternary methyl ammonium cartridge and diluted with saline for subsequent *in vitro* and *in vivo* experiments.

### 2.8. Synthesis of [^89^Zr]Zr-MXene

A defined volume (100 µL) of ^89^Zr-oxalate solution (corresponding to 1.0-6.0 mCi, 37-222 MBq) was transferred into a metal-free vial, followed by the addition of 200 µL of 1 M oxalic acid. The pH of the solution was adjusted to 7.0-7.5 using 1 M Na_2_CO_3_, initially by adding 250 µL of base, followed by smaller aliquots (< 10 µL) until the target pH was reached. The solution was then incubated for 3 min at room temperature, after which 300 µL of 0.5 M HEPES buffer was added to neutralize the solution. The pH-adjusted ^89^Zr solution was then added to the MXene-APTES-DFO dispersion (1.5 mg in 200 µL of 0.5 M HEPES buffer, pH 7.5), maintaining the pH within 7.0-7.5 range. The mixture was gently agitated at 350 rpm for 60 min at room temperature. Radiolabeling efficiency was monitored using radio-iTLC with 50 mM diethylenetriaminepentaacetic acid (DTPA) (pH 5.5) as the mobile phase. Bound ^89^Zr appeared at the origin (R_f_ ≈ 0.2), while free ^89^Zr was chelated by DTPA and migrated with the solvent front (R_f_ > 0.9). Radiolabeling yield was calculated by integrating the area under the curve corresponding to R_f_ 0.1-0.2 and dividing by the total area, multiplied by 100. Upon completion, the reaction was quenched with 5 µL of 50 mM DTPA, and unbound ^89^Zr was removed by centrifugation followed by purification using a 100 kDa molecular weight cutoff filter. The radiochemical purity of [^89^Zr]Zr-MXene was subsequently assessed by radio-iTLC 40-41.

### 2.9. *In Vitro* Stability Analysis of [^89^Zr]Zr-MXene

The *in vitro* serum stability of [^89^Zr]Zr-MXene was evaluated by incubating 100 μL of the complex with 100 μL of 2× whole mouse serum, human serum, or saline at 37 °C under continuous shaking (550 rpm) for up to 7 days. At predetermined time points, the supernatant was analyzed using radio-iTLC. Results were expressed as the percentage of intact [^89^Zr]Zr-MXene over time (n = 5).

### 2.10. *In Vivo* PET/CT Images of [^89^Zr]Zr-MXene

For PET imaging, [^89^Zr]Zr-MXene (0.814 MBq/100μL, 0.9% saline) was intravenously injected into healthy BALB/c mice (n = 4). PET scans were acquired at multiple time points post-injection, ranging from 1 h to 21 days, using small-animal PET/CT scanner (nanoScan, Mediso Medical Imaging Systems). Each PET scan was performed for 20 min and reconstructed with a three-dimensional ordered-subset expectation maximization algorithm (four iterations, six subsets). Micro-CT imaging (50 kVp, 0.16 mAs) was conducted immediately after each PET scan for attenuation correction and anatomical reference. Quantitative PET imaging analysis involved manually drawing regions of interest (ROIs) over the lungs, liver, and spleen on reconstructed PET images at each timepoint (0.5, 1, 2, 4, 7, 10, 14, and 21-days post-injection). SUV_max_ values were extracted for each organ at each timepoint. Data are presented as mean ± SD (n = 4 per group). Time-activity curves were generated by plotting mean SUV_max_ against time for each organ, showing the uptake and clearance profile over the study period.

### 2.11. Noncompartmental Pharmacokinetic Analysis of [^89^Zr]Zr-MXene

Noncompartmental pharmacokinetic analysis was performed on the organ ROI data described above. For each animal, the area under the SUVmax-time curve (AUC) was calculated for the lungs, liver, and spleen using the linear trapezoidal rule, summing the areas of consecutive time intervals from day 0 to day 21 (AUC(0-21d)). AUC was calculated individually for each animal, then averaged across animals within each organ group (n = 4) to obtain the mean ± SD reported for each organ. Extrapolation to AUC(0-∞) was not performed, since the terminal timepoints did not consistently reach a stable log-linear elimination phase across all organs. AUC(0-21d) is therefore reported as the observed exposure metric, consistent with standard noncompartmental practice under these conditions.

### 2.12. Blood Clearance study of [^89^Zr]Zr-MXene

To characterize the systemic pharmacokinetics of the tracer, we measured blood radioactivity concentration over time following intravenous injection. Blood samples were collected at 0.25, 0.5, 1, 2, 4, 8, 12, and 24 h post-injection (n = 3 per timepoint), and radioactivity was normalized to the injected dose and expressed as percent injected dose per gram of blood (%ID/g). Blood pharmacokinetic parameters were derived by noncompartmental analysis. The initial blood half-life was estimated from the terminal slope of the early distribution phase. The area under the blood concentration time curve to the last measured timepoint, AUC(0-24h), was calculated by the linear trapezoidal method. AUC(0-24h) was reported as the primary measure of blood exposure, in line with standard practice when full terminal-phase kinetics aren't captured. This blood clearance analysis was performed alongside quantitative PET region-of-interest (ROI) analysis and organ-level AUC calculations to give a complete pharmacokinetic picture of the tracer.

### 2.13. *Ex Vivo* Biodistribution Analysis of [^89^Zr]Zr-MXene

To evaluate the biodistribution and potential long-term toxicity of [^89^Zr]Zr-MXene, healthy ICR mice (n = 4 per group; 24 mice in total) were intravenously injected with [^89^Zr]Zr-MXene (0.185 MBq in 100 μL of 0.9% saline). For the day-0 group, blood and organs were collected immediately after injection to establish the initial distribution of the radiolabeled material. For the remaining groups, samples were collected at predetermined time points of 1, 4, 7, 14, and 21 days post-injection. At each time point, mice were euthanized, and blood and organs of interest, including muscle, intestine, stomach, kidneys, spleen, liver, lungs, heart, and bone, were harvested. The radioactivity in each tissue and organ was measured using a gamma counter, and uptake was expressed as the percentage of injected dose per gram of tissue (%ID/g).

### 2.14. Histopathological Analysis

Histopathological evaluation was performed to assess potential tissue-level toxicity associated with *in vivo* administration of [^89^Zr]Zr-MXene. At 21^st^ day post injection, mice were euthanized, and histopathological analysis of major organs including the liver, lungs, and spleen was performed according to standard laboratory procedures. Stained sections were examined under light microscope for evidence of inflammation, necrosis, fibrosis, or other pathological abnormalities, and compared with corresponding tissues from control animals.

## 3. Results and Discussion

### 3.1. Results of Synthesis and Characterization

MXenes, particularly Ti_3_C_2_T_x_, are distinctive 2D materials characterized by ultrathin layers, large surface area, and abundant surface terminal groups T_x_ (-O, -OH, -F) 42. These features confer strong hydrophilicity, excellent dispersibility, tunable surface chemistry, and high electrical conductivity, thereby enabling broad biomedical applications. MXenes have been investigated for improved biocompatibility, stability, size control, and functional performance, enabling their exploration in photothermal therapy, antimicrobial treatments, biosensing, imaging, drug delivery, and theranostic applications 43-46. Understanding the long-term *in vivo* behavior of MXene nanosheets is important for evaluating their potential as drug delivery and theranostic platforms. Here, we combine radiolabeling, PET/CT imaging, and quantitative organ analysis to map biodistribution, clearance, and potential toxicity over 21 days.

To enable *in vivo* tracking of MXene (Ti_3_C_2_T_x_), we synthesized Ti_3_C_2_T_x_ from the MAX phase (Ti_3_AlC_2_). The successful etching of the aluminum (Al) layer was initially indicated by a color change from gray to black, and further confirmed by X-ray diffraction (XRD) analysis. The characteristic (002) peak of Ti_3_AlC_2_ shifted to lower 2θ values (Figure 2a), reflecting increased interlayer spacing due to selective Al removal 47-49. Peak broadening and decreased intensity further indicated exfoliation and increased structural disorder. The disappearance of the peak near 40° confirmed the effective removal of the Al layer, as already reported. FTIR spectroscopy of the MXene revealed broad O-H stretching around 3440 cm⁻¹ and characteristic Ti-O/Ti-O-C absorptions in the 550-700 cm⁻¹ region, confirming surface terminations with -OH and -O groups as shown in the supplementary document (Figure S1) 50.

X-ray photoelectron spectroscopy (XPS) analysis revealed changes in the surface chemical states of MXene nanosheets before and after chemical modification. As shown in the survey scan of the pristine MXene nanosheets (red line) (Figure 2b), signals corresponding to the elements Ti, carbon (C), fluorine (F), and oxygen (O) were detected, indicating that surface functional groups such as -OH and-F were introduced during the etching process and the data is presented in supplementary information (Table S1 and S2) 51.

The SEM image of Ti_3_AlC_2_ (Figure 2c) shows a well-ordered layered architecture with densely packed, smooth lamellar surfaces characteristic of its compact crystalline morphology. Upon selective etching and subsequent exfoliation, the material was transformed into MXene (Figure 2d), displaying a markedly delaminated and porous framework composed of ultrathin, sharp-edged nanosheets. This morphological evolution clearly evidences the successful removal of Al layers and the effective expansion of interlayer spacing, confirming the formation of few-layered 2D MXene sheets 52.

Subsequently, the delaminated MXene with surface-terminating groups was functionalized with APTES to yield MXene-APTES Supplementary material (Scheme S1). Successful surface functionalization was evidenced by a shift of the (002) XRD peak from 6.89° for MXene to 4.04° for MXene-APTES (Figure 2a), consistent with interlayer expansion upon grafting of -NH_2_ groups 53.

FTIR spectroscopy was further employed to confirm surface modification. After silanization with APTES, new absorption bands appeared at around 2841-2952 cm⁻¹, corresponding to the C-H stretching vibrations of the propyl chains. Slight broadening in the N-H/O-H region around 3422 cm⁻¹ indicated the presence of surface amine and hydroxyl groups (Figure S1). A new peak at 1458 cm⁻¹ was attributed to the C-N stretching vibration, while an enhanced feature near 1378 cm⁻¹ corresponded to Si-O-Si/Si-O-Ti vibrations, collectively confirming covalent attachment of silane molecules to the MXene surface 54.

XPS analysis of MXene-APTES (blue line) further confirmed successful grafting. Compared to pristine MXene (red line) in Figure 2b, two additional peaks at 400 eV and 102 eV, corresponding to nitrogen (N) 1s and silicon (Si) 2p, respectively, were observed for MXene-APTES (blue line) (Figure 2b; Tables S1 and S2). The presence of N 1s and Si 2p signals in the survey spectra of MXene-APTES clearly demonstrates the successful incorporation of N and Si 55. Together with the XRD and FTIR results, these results further confirm that APTES was successfully bonded to the MXene surface. Following that, MXene-APTES was conjugated with the *p*-SCN-Bz-DFO chelator to provide suitable sites for ^89^Zr chelation (Scheme S2).

FTIR analysis of the conjugated product (Figure S1) revealed that upon reaction with *p*-SCN-Bz-DFO, the characteristic isothiocyanate (NCS) absorption band near 2100 cm⁻¹ observed in the spectrum of commercial *p*-SCN-Bz-DFO disappeared, indicating successful reaction between the -NCS group and surface -NH₂ groups on MXene-APTES. Concurrently, a new band appeared at 1554 cm⁻¹, corresponding to N-H bending and C-N stretching vibrations of the newly formed thiourea linkage. Additional peaks observed at 1614, 1554, and 1497 cm⁻¹ were attributed to C=C stretching of the aromatic backbone, while the band at 3307 cm⁻¹ corresponded to N-H stretching of a secondary amide, consistent with those of commercial *p*-NCS-Bz-DFO 56. These spectral evolutions collectively confirm successful covalent conjugation of *p*-SCN-Bz-DFO to MXene-APTES via amine-isothiocyanate coupling.

The conjugation of* p*-SCN-Bz-DFO to MXene-APTES was further validated by XPS analysis (green line, Figure 2b). A slight decrease in the Ti 2p signal intensity indicated surface coverage by the DFO layer. Additionally, a new sulfur (S) 2p peak appeared at 161 eV, confirming the successful introduction of sulfur originating from the thiourea linkage of DFO. The nitrogen (N) 1s peak broadened with a new component near 399 eV, corresponding to amide or thiourea nitrogen formed during the reaction between APTES amine groups and the isothiocyanate group of *p*-SCN-Bz-DFO chelator. The C 1s spectrum also showed an increased intensity at 284.0 eV due to C=O bonding, further supporting successful conjugation of DFO onto the MXene-APTES surface (Table. S1 and S2).

Morphological evaluation using SEM and transmission electron microscopy (TEM) further corroborated the structural integrity of the MXene. The SEM image of MXene-APTES-DFO (Figure 2e) revealed thin, few-layered sheets with wrinkled surfaces, indicative of successful conjugation without compromising the intrinsic layered morphology. TEM analysis (Figure 2f) displayed the characteristic flake-like structure of MXene-APTES-DFO, demonstrating that the structural integrity of MXene remained intact after *p*-SCN-Bz-DFO chelator conjugation. The selected area electron diffraction pattern (inset) exhibited sharp diffraction spots, confirming the highly crystalline nature of the material. Furthermore, high-resolution TEM imaging coupled with energy-dispersive X-ray spectroscopy (EDS) elemental mapping (Figure 2g, 2h & S2) displayed uniform distributions of Ti, C, O, N, Si, and S for each successive step. The clear presence of Si, N, and S peaks in the EDX spectra of MXene-APTES-DFO strongly supports the successful surface modification of MXene via sequential APTES functionalization and subsequent *p*-SCN-Bz-DFO chelator attachment.

DLS analysis demonstrated that MXene and MXene-APTES-DFO nanosheets maintained good colloidal stability in aqueous suspension (pH 7.3), with average hydrodynamic diameters of 277 ± 56 nm and 300 ± 55 nm, respectively, and a low polydispersity index (PDI) of approximately 0.2 (Figure 3a). To further evaluate formulation stability under physiologically relevant conditions, additional DLS measurements were performed in the final injection vehicle (0.9% normal saline) and after incubation in 90% serum for 24 h. No significant changes in hydrodynamic diameter or PDI were observed under either condition, confirming excellent colloidal stability and suggesting that substantial particle aggregation was unlikely (Table S5).

Zeta potential analysis revealed a shift from -24.08 ± 1.65 mV for MXene to 14.32 ± 2.63 mV for MXene-APTES, indicating successful surface amination and improved aqueous dispersibility 57. After conjugation with the *p*-SCN-Bz-DFO chelator, the zeta potential slightly shifted to -28.93 ± 1.94 mV, suggesting that surface modification induced only a marginal change in surface charge (Figure 3b, Table S3). Such a narrow variation implies that the *p*-SCN-Bz-DFO chelator functionalization did not significantly affect the colloidal behavior or expected *in vivo* biodistribution of the MXene nanosheets.

### 3.2. Results of *In Vitro* Cytotoxicity

Following successful synthesis and characterization, the cytotoxicity of MXene-APTES-DFO was evaluated across four cell lines: MRC-5, A549, Detroit-551, and A375. Cell viability was measured after exposure to increasing concentrations of MXene-APTES-DFO (0-0.9 mg/mL) (Figure 3c). Among the tested cell lines, Detroit-551 cells showed no significant toxicity, maintaining cell viability above 88.01 ± 2.55% across the entire concentration range. A dose-dependent decrease in viability was observed in all other cell lines, with the strongest cytotoxic effects in A549 (61.77 ± 2.97%), whereas the normal cell lines exhibited relatively higher resistance. Overall, cancerous cells were more susceptible to MXene-APTES-DFO than normal cells, likely due to differences in metabolic activity, consistent with previous reports 37.

### 3.3. Results of Radiosynthesis

MXene-APTES-DFO was successfully radiolabeled with ^89^Zr following established protocols 58,59. (Figure 4a). Radiolabeling efficiency of the reaction was monitored by instant thin-layer chromatography (iTLC) using 50 mM DTPA, with bound ^89^Zr appearing at the origin (retention factor [R_f_] ≈ 0.2) and free ^89^Zr migrating with the solvent front (Rf > 0.9). The reaction was quenched with 5 µL of 50 mM DTPA, and the crude radiolabeled product was purified using an Amicon Ultra 100 kDa centrifugal filter. An overall radiolabeling yield of > 95% was achieved, and the purified [^89^Zr]Zr-MXene exhibited a radiochemical purity of 99.39 ± 0.66% (n = 5) (Figure 4b).

To rule out nonspecific radiolabeling of MXene nanosheets with ^89^Zr, MXene-APTES without a DFO chelator was subjected to the same radiolabeling procedure. Radio-iTLC analysis showed that ^89^Zr did not bind to MXene in the absence of the chelator, indicating that the DFO chelator is essential for stable conjugation and coordination of ^89^Zr to MXene nanosheets. These results demonstrate that stable radiolabeling of MXene requires DFO chelation and that nonspecific adsorption of ^89^Zr onto the MXene surface is negligible under the experimental conditions (Figure S3).

### 3.4. Results of *In Vitro* Stability

Before starting *in vivo* studies, the *in vitro* serum stability of [^89^Zr]Zr-MXene was assessed in mouse serum, human serum, and 0.9% sodium chloride over 7 days. Stability was quantified using iTLC (n = 5; Figure S4), and the results are summarized in Figure 4c. The data indicate negligible release of ^89^Zr from [^89^Zr]Zr-MXene throughout the 7-day incubation period, with marginally higher stability observed in mouse serum (96.0 ± 1.8%) and human serum (97.3 ± 1.9%) relative to saline (95.66 ± 3.4%) on the 7^th^ day of incubation.

### 3.5. Results of *In Vivo* PET/CT Imaging

The *in vivo* pharmacokinetic profile of [^89^Zr]Zr-MXene was examined using PET/CT imaging in healthy BALB/c mice following intravenous administration. Each mouse received 0.814 MBq of [^89^Zr]Zr-MXene containing 5 µg of MXene-APTES-DFO dispersed in sterile 0.9% normal saline. Based on an average body weight of 22 g, the administered MXene dose corresponded to approximately 0.23 mg/kg. The radiolabeled formulation was prepared with a radiochemical yield of 95% (n = 5) and a radiochemical purity exceeding 99.39 ± 0.66% (n = 5), corresponding to a final specific activity of approximately 141 MBq/mg [^89^Zr]Zr-MXene (Table S6). The same radiolabeled formulation and comparable injected MXene dose were used for both longitudinal PET imaging and *ex vivo* biodistribution studies. PET/CT scans were acquired using a Mediso system over 21 days, corresponding to approximately seven half-lives of ^89^Zr. Maximum intensity projection (MIP) images revealed strong initial pulmonary accumulation followed by redistribution to the liver and spleen, consistent with uptake by the mononuclear phagocyte system (MPS) (Figure 5a; S5). Prominent gallbladder activity was observed during the early phase (1-8 h), which gradually diminished over time, whereas hepatic retention remained relatively constant (Figure S5). Minimal bone uptake was observed throughout the imaging period, supporting the *in vivo* stability of the DFO-mediated ^89^Zr chelation under the experimental conditions. PET ROI analysis showed a time-dependent decline in tracer uptake across the lungs, liver, and spleen (Figure 5b). At the earliest timepoint (0.5 d), SUVmax values were comparable across organs: 6.69 ± 0.6 in liver, 6.64 ± 0.74 in spleen, and 6.10 ± 0.79 in lungs. Liver uptake peaked at day 1 (SUVmax = 7.50 ± 1.21), then declined progressively through day 21 (SUVmax = 1.63 ± 0.27). Spleen uptake followed a similar pattern, dropping from 6.64 ± 0.74 at day 0.5 to 1.04 ± 0.52 at day 21. Lung uptake was the lowest of the three organs at every timepoint, falling from 6.10 ± 0.79 at day 0.5 to 1.23 ± 0.24 by day 10 and staying at 0.89 ± 0.10 through day 21. By day 21, SUVmax values had converged across all three organs, indicating substantial clearance from the reticuloendothelial system over the 21-day observation period. Noncompartmental AUC (0-21d) values, calculated per animal using the linear trapezoidal method and expressed as mean ± SD across animals (n = 4), differed across organs (Figure 5c). Liver showed the highest overall exposure (AUC = 67.61 ± 6.92), followed by spleen (58.33 ± 5.32) and lungs (46.27 ± 3.38). This ranking mirrors the peak SUVmax values seen in the time-activity curves: the liver's higher initial and sustained uptake translated into the greatest cumulative exposure over the 21-day observation period.

The higher AUC in liver and spleen relative to lungs fits their central role in reticuloendothelial system (RES)-mediated clearance of intravenously administered nanomaterials. These organs are rich in phagocytic Kupffer cells (liver) and macrophages (spleen), which typically sequester circulating nanoparticles more extensively and over a longer duration than the lungs. Overall, the organ-ranked AUC pattern (liver > spleen > lungs) matches the expected biodistribution and clearance profile for nanomaterials cleared primarily through the RES, and quantitatively confirms the trends already visible in the time-activity curves. Blood radioactivity declined rapidly following intravenous injection, from 5.76 ± 0.70 %ID/g at 0.25 h to 3.80 ± 0.53 %ID/g at 0.5 h and 1.01 ± 0.65 %ID/g by 1 h, consistent with a fast initial half-life of approximately 0.26 h. Beyond 2 h, blood levels declined further but stayed low and fairly stable — 0.23-0.20 %ID/g between 2-8 h, dropping to 0.085 %ID/g by 24 h (Figure 5d). This pattern points to near-complete clearance of the tracer from systemic circulation within the first 1-2 h post-injection.

Noncompartmental analysis of the blood time activity curve gave an AUC(0-24h) of 5.45 ± 0.41 %ID/g·h. This rapid blood clearance fits with prompt sequestration of the nanoparticle by the reticuloendothelial system, matching the early and sustained uptake we saw in the liver and spleen ROI data. Taken together, the blood clearance kinetics and organ-level ROI/AUC data describe the systemic distribution and elimination behavior of the MXene *in vivo*.

### 3.6. Results of *Ex Vivo* Biodistribution

To further investigate the tissue biodistribution and pharmacokinetic profile of MXene, [^89^Zr]Zr-MXene (0.185 MBq/100 µL 0.9% saline) was administered intravenously to mice via the tail vein. Mice were sacrificed at predetermined time points post-injection, and major organs were excised to quantify radioactivity using a gamma counter; uptake was reported as the percentage of injected dose per gram of tissue (%ID/g). It was found that immediately after administration (day 0), the lungs exhibited the highest uptake (30.43 ± 5.39 %ID/g), consistent with initial pulmonary sequestration, which may be influenced by the lateral dimensions of the nanosheets and first-pass passage through the pulmonary capillary network. This pulmonary accumulation decreased rapidly to 13.30 ± 4.96 %ID/g by day 1 and continued declining over 21 days, reaching 3.86 ± 0.19 %ID/g, indicating progressive clearance from the lungs. The liver and spleen demonstrated significant retention of [^89^Zr]Zr-MXene throughout the study period, reflecting active uptake by the reticuloendothelial system (RES). Liver accumulation increased from 9.48 ± 3.77 %ID/g at day 0 to a peak of 30.16 ± 5.42 %ID/g on day 7, followed by a gradual decline to 8.13 ± 0.72 %ID/g by day 21. Similarly, the spleen exhibited progressive accumulation from 1.61 ± 0.61 %ID/g at day 0 to 9.31 ± 3.17 %ID/g on day 7, decreasing slightly to 4.99 ± 0.62 %ID/g by day 21, consistent with the PET/CT images. These temporal trends are consistent with the known role of the liver and spleen in the sequestration and processing of circulating nanomaterials by the reticuloendothelial system. Other organs displayed comparatively lower uptake. The kidneys displayed moderate early accumulation (2.82 ± 0.59 %ID/g at day 0), which decreased rapidly within the first week, indicating limited renal retention (Table S4). Blood activity remained low throughout the study, increasing slightly on day 1 (0.74 ± 0.88 %ID/g) before declining to negligible levels (0.04 ± 0.01 %ID/g by day 21), consistent with rapid distribution from the circulation into tissues. Heart and intestinal tissues exhibited minimal accumulation at all time points (< 1 %ID/g) (Figure 6a-6b). These findings are consistent with sequestration of MXene nanosheets by the reticuloendothelial system, which has been widely reported for nanomaterials of comparable physicochemical characteristics.

Since free ^89^Zr is known to be an osteophilic cation with rapid and persistent bone accumulation, its detection in bones serves as a reliable indicator of radiolabel instability 60,61. Mice injected with [^89^Zr]Zr-MXene exhibited remarkable *in vivo* stability over 3 weeks, with only 1.04 ± 0.14 %ID/g bone uptake detected at day 21 post-injection. This negligible bone accumulation confirmed the strong chelation stability of the tracer, and any minor residual uptake may be attributed to intrinsically adsorbed, rather than chelator-bound ^89^Zr on the MXene surface. The minimal bone uptake of [^89^Zr]Zr-MXene (<1.04 %ID/g at 21 days) provides strong evidence for the excellent *in vivo* stability of the ^89^Zr-DFO chelate, since substantial release of free ^89^Zr would be expected to result in increased skeletal accumulation. This stability supports the reliability of PET imaging for monitoring the biodistribution and retention of the radiolabeled MXene construct over the study period. However, an important distinction should be made between the radiometal chelate and the inorganic MXene framework. PET detects the distribution of ^89^Zr and therefore primarily reports the fate of the DFO-associated radiolabeled species rather than the Ti_3_C_2_T_x_ lattice itself. Consequently, the observed PET signal cannot unequivocally distinguish intact MXene nanosheets from smaller DFO-labeled degradation fragments that may be generated during gradual *in vivo* degradation of the nanomaterial.

This limitation is particularly relevant for evaluating the long-term fate and biodegradation of MXene. In principle, cleavage or degradation of the Ti_3_C_2_T_x_ framework could occur without immediate loss of the ^89^Zr-DFO label, allowing radiolabeled degradation products to remain PET-visible. Conversely, loss or redistribution of the radiolabel could occur independently of degradation of the Ti-containing framework. Therefore, PET biodistribution should be interpreted as the distribution of the radiolabeled construct rather than a direct measurement of the intact MXene core. The present findings thus provide reliable information on the biodistribution and pharmacokinetic behavior of the ^89^Zr-DFO-labeled construct, while the chemical fate of the MXene framework remains to be independently established.

Future studies combining longitudinal PET imaging with complementary elemental analysis, particularly ICP-MS for titanium, could provide orthogonal assessment of the fate of the MXene framework *in vivo*. Quantification of Ti concentrations in blood and individual organs at corresponding time points would enable direct comparison of the temporal distribution of the inorganic MXene component with the distribution of the ^89^Zr radiolabel. Such a PET-ICP-MS approach could help determine whether changes in PET signal are accompanied by corresponding changes in Ti distribution and could provide evidence for the retention, redistribution, or clearance of the Ti_3_C_2_T_x_ framework. Importantly, this complementary analysis would allow future studies to better estimate the extent and kinetics of MXene degradation and to distinguish persistence of the inorganic framework from persistence of radiolabeled DFO-containing species. Together, radiotracer-based imaging and elemental Ti analysis would therefore provide a more comprehensive assessment of both the pharmacokinetics of the labeled construct and the chemical fate of the MXene nanosheets.

In an experiment [^89^Zr]Zr-DFO was also injected into the mice however rapid clearance from body was observed within 60 min post injection. The predominant accumulation of MXene in the liver, lungs, and spleen aligns with previous reports. However, earlier studies using Ti quantification by ICP-MS reported comparatively higher apparent accumulation of MXene in the blood and heart 25. In contrast, the radiolabeling approach employed in this study enables highly sensitive, quantitative, and longitudinal non-invasive evaluation of the biodistribution and pharmacokinetics of the radiolabeled MXene construct. When combined with complementary elemental techniques such as ICP-MS, this strategy may further strengthen the assessment of the *in vivo* fate of MXene nanomaterials. To investigate whether the pronounced pulmonary accumulation could result from particle aggregation or formulation instability, additional DLS measurements were performed using the final injection vehicle (0.9% normal saline) and after 24 h incubation in 90% serum. No significant changes in hydrodynamic diameter or polydispersity index (PDI) were observed compared with the aqueous suspension (Table S5), demonstrating excellent colloidal stability under both formulation and physiologically relevant conditions. These findings suggest that extensive aggregation prior to injection or serum-induced aggregation is unlikely to account for the observed pulmonary retention. Instead, the high lung uptake is more likely related to the intrinsic biological behavior of the two-dimensional MXene nanosheets, including their lateral dimensions, surface characteristics, and interactions with the pulmonary microvasculature.

To further elucidate the excretion pathways, mice were placed in metabolic cages and, following intravenous administration of [^89^Zr]Zr-MXene (0.185 MBq/100 µL water), urine and feces were collected over 7 days post-injection (Figure 6c). Renal excretion predominated during the early phase, with the highest elimination occurring within the first 24 h. In contrast, fecal excretion, reflective of hepatobiliary elimination, was initially low but increased after day 2, suggesting a delayed contribution from biliary clearance. By day 7, both urinary and fecal excretion demonstrated that renal and hepatobiliary pathways contributed to tracer elimination. However, measurable activity remained in the liver, spleen, and lungs at later time points, indicating that complete systemic clearance had not yet been achieved during the study period.

Renal and hepatobiliary elimination are recognized as important clearance pathways for inorganic nanomaterials, with their relative contributions influenced by particle size, geometry, surface chemistry, and biodegradation. Because the intact MXene-APTES-DFO nanosheets used in this study exhibited an average hydrodynamic diameter of approximately 300 nm, direct glomerular filtration of the intact nanosheets is unlikely. Nevertheless, the urinary excretion of radioactivity observed in this study suggests that renal elimination may occur following the gradual *in vivo* degradation of MXene into smaller fragments or partially soluble degradation products, as reported previously for Ti_3_C_2_T_x_ MXene 62,63. In contrast, larger MXene fragments or those internalized by RES macrophages are more likely to undergo biliary clearance into the feces. Accordingly, renal and hepatobiliary dual clearance suggests that both excretory pathways contribute to the elimination of MXene-derived materials, with their relative contributions likely influenced by particle size and surface properties. In the present study, urinary and fecal radioactivity was quantified as %ID/g to characterize the temporal profile of the elimination pathways. Radioactivity in both urine and feces progressively decreased and fell below the detection limit after day 7. However, measurable activity remained in the liver, spleen, and lungs at later time points, indicating persistent organ retention despite the absence of detectable radioactivity in excreta. Because cumulative urine and feces collections were not performed, a quantitative whole-body mass balance could not be established. Future studies incorporating cumulative recovery of the injected dose together with direct characterization of degradation products will provide a more comprehensive assessment of the clearance kinetics of MXene nanosheets. Although gradual oxidative degradation of MXene under physiological conditions has been reported to generate partially soluble or ultrafine degradation products 64,65, direct evidence for the formation of such degradation products was not obtained in the present study. Therefore, this proposed mechanism should be considered a plausible explanation for the observed renal radioactivity rather than a demonstrated clearance pathway.

Given that non-biodegradable or slowly degradable nanomaterials may pose risks associated with prolonged tissue retention, understanding the degradation kinetics and elimination pathways of MXene remains important for its safe biomedical application. Although previous studies have reported no significant acute *in vivo* toxicity of MXene-based systems, their long-term hepatic and pulmonary retention warrants further investigation. Histopathological analysis was performed to evaluate potential tissue morphological changes associated with the prolonged retention of [^89^Zr]Zr-MXene. At day 21 post-injection, hematoxylin and eosin-stained sections of the liver, lung, and spleen showed preserved histological architecture comparable to saline-treated controls, with no evidence of necrosis or fibrosis. These findings suggest that prolonged retention of [^89^Zr]Zr-MXene was not associated with detectable histopathological alterations in the examined organs under the experimental conditions of this study, consistent with previous reports 25.

By integrating quantitative pharmacokinetic analysis, biodistribution, organ retention, histopathological evaluation, and clearance studies, the present work provides a framework for the rational design of MXene-based drug delivery and theranostic platforms. Systemic optimization of nanosheet size, surface chemistry, and surface functionalization may improve biodistribution and clearance while preserving the desired physicochemical properties. Histopathological examination of the liver, lungs, and spleen at 21 days following administration of 5 µg of MXene-APTES-DFO (approximately 0.23 mg/kg) revealed no obvious morphological abnormalities under the experimental conditions of this study. Future investigations incorporating comprehensive toxicological assessments, including serum biochemistry, hematology, inflammatory biomarkers, quantitative histopathological scoring, multi-isotopic labeling, and extended observation periods, will be important for further evaluating the long-term biological fate and safety profile of MXene nanomaterials. In addition, evaluation of tumor-targeted MXene constructs in relevant disease models will further advance the preclinical development of MXene-based biomedical platforms.

## Conclusion

This study provides a quantitative *in vivo* pharmacokinetic profile of [^89^Zr]Zr-MXene nanosheets, enabling longitudinal tracking of biodistribution and clearance over 21 days. Following systemic administration, MXene showed rapid distribution with transient accumulation in the lungs, liver, and spleen, followed by gradual elimination through both renal and hepatobiliary pathways. Minimal bone uptake supported *the in vivo* stability of the radiolabel, while histopathological examination of the liver, lungs, and spleen at 21 days post-injection revealed preserved tissue architecture with no obvious morphological abnormalities under the experimental conditions.

Overall, these findings provide quantitative insight into the biodistribution, pharmacokinetics, clearance pathways, and *in vivo* stability of radiolabeled MXene nanosheets. This work establishes a framework for evaluating the *in vivo* behavior of MXene-based nanomaterials and may facilitate the rational design of MXene-based nanocarriers. Furthermore, the findings highlight the importance of controlling nanosheet size and surface chemistry to optimize biodistribution and clearance. Comprehensive long-term toxicological studies will be required to further evaluate the biosafety of MXene nanomaterials prior to clinical translation.

## Supplementary Material

Supplementary figures and tables.

## Figures and Tables

**Figure 1:**
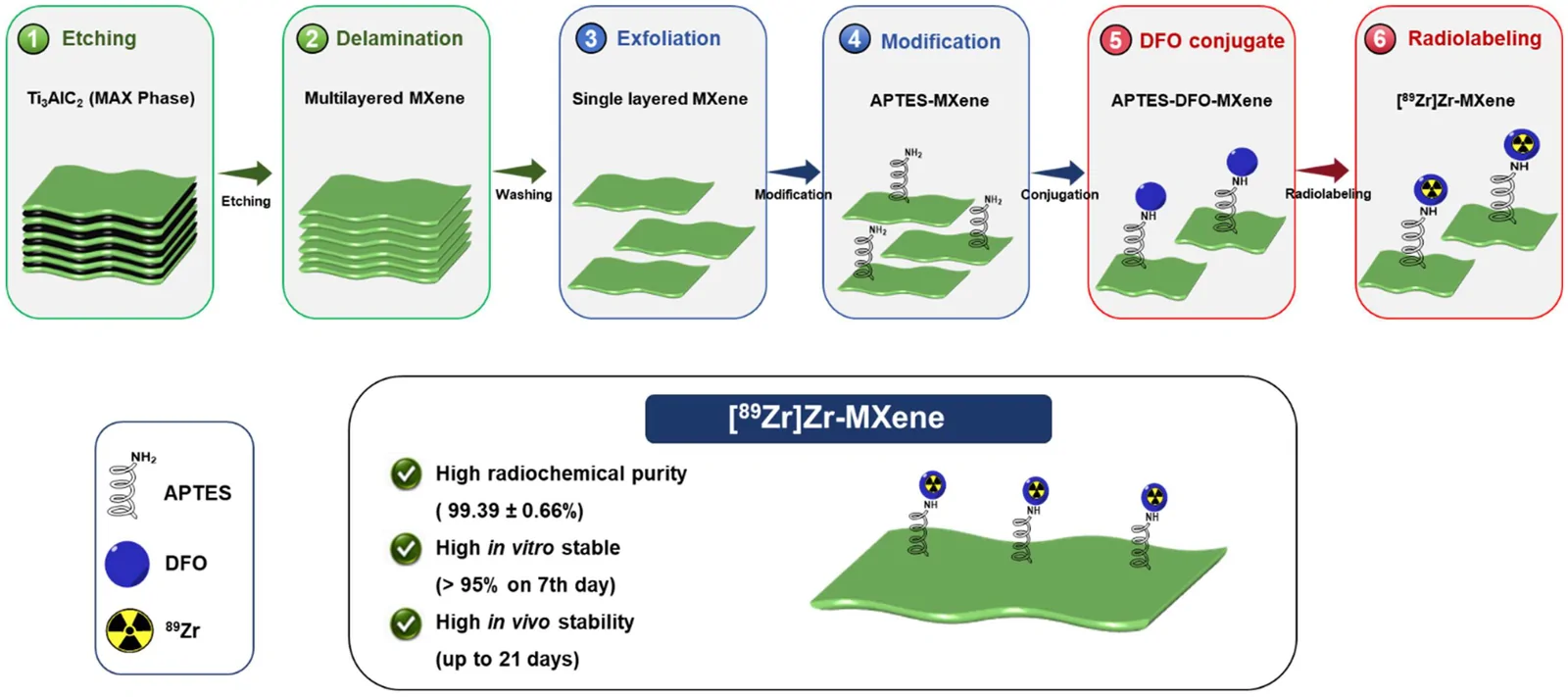
Synthesis Schematic. Overview of the preparation of [^89^Zr]Zr-MXene beginning with the MAX phase (Ti₃AlC₂) etching, followed by washing, surface functionalization of MXene using (3-aminopropyl)triethoxysilane (APTES) and the *p*-isothiocyanatobenzyl desferrioxamine (*p*-SCN-Bz-DFO) chelator, and subsequent radiolabeling with Zirconium-89 (^89^Zr).

**Figure 2:**
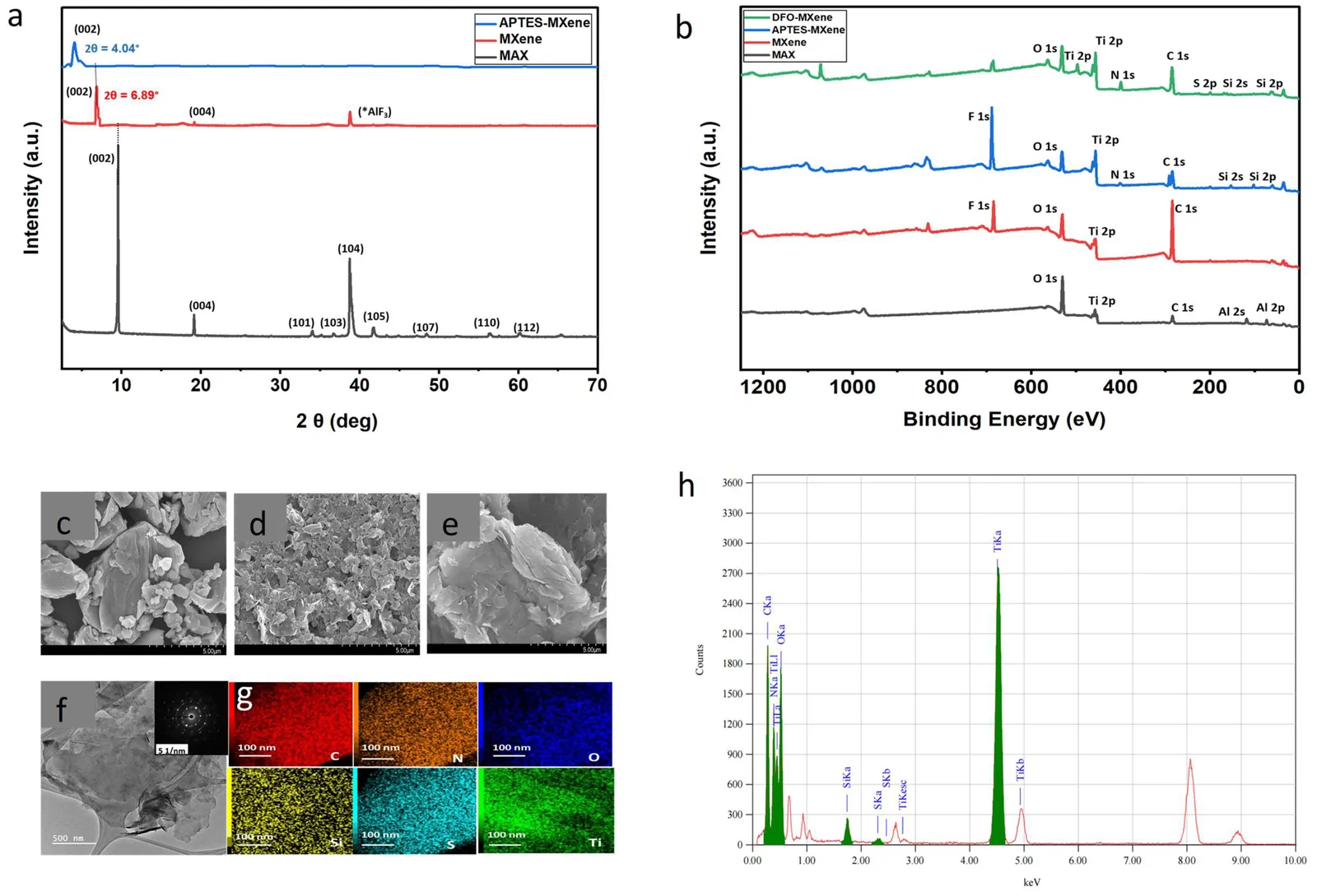
Characterization of materials. (a) X-ray diffraction (XRD) analysis; (b) X-ray photoelectron spectroscopy (XPS) analysis of the MAX phase, MXene, MXene functionalized with (3-aminopropyl)triethoxysilane (MXene-APTES), and MXene-APTES conjugated with *p*-isothiocyanatobenzyl-desferrioxamine (MXene-APTES-DFO); (c-e) SEM images of the MAX phase, MXene and MXene-APTES-DFO, respectively; and (f) Transmission electron microscopy (TEM) images of MXene-APTES-DFO with corresponding (g,h) elemental mapping and EDX spectra. (inset shows the selected area electron diffraction [SAED] pattern).

**Figure 3:**
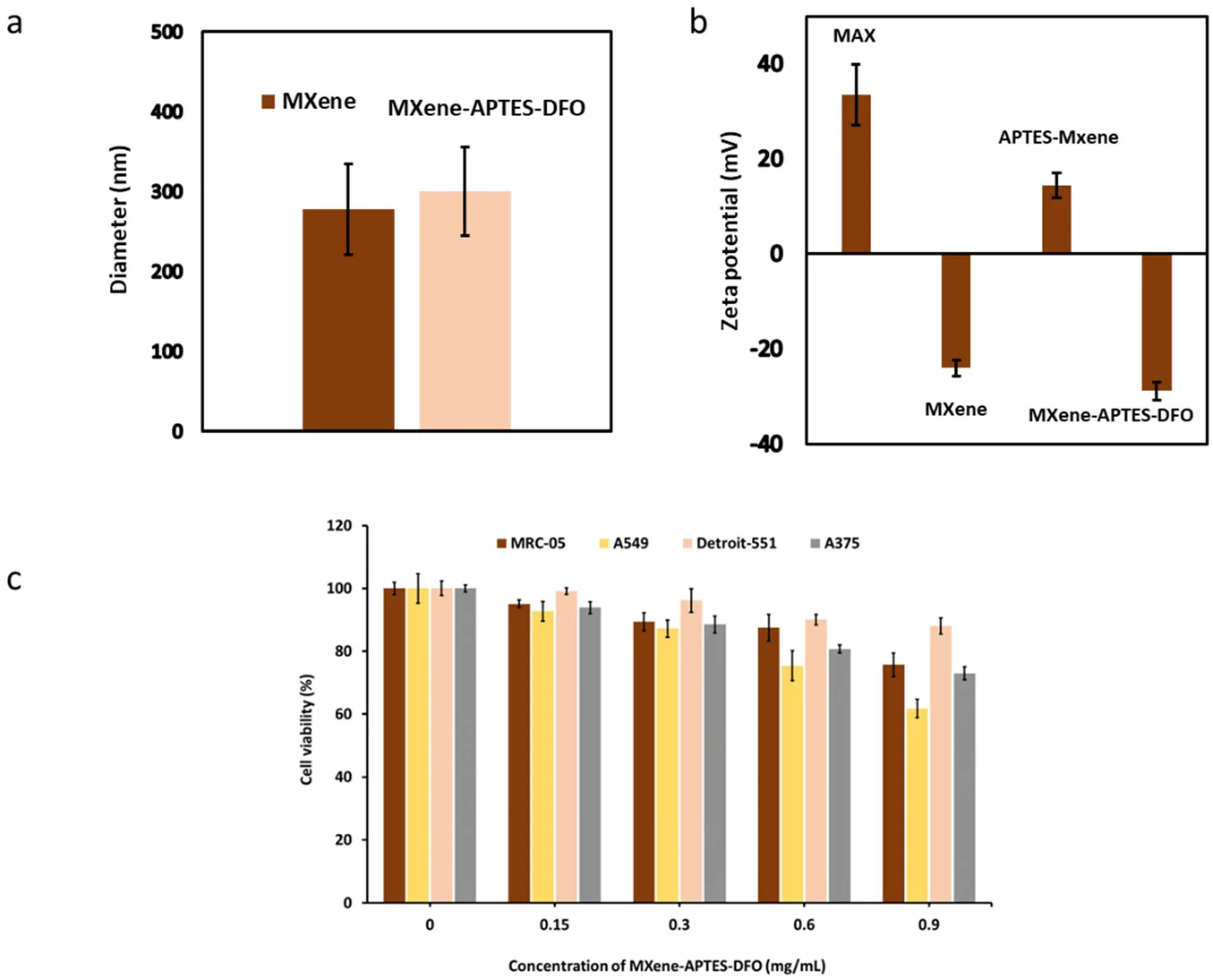
Characterization and *in vitro* cytotoxicity of MXene nanosheets. (a) DLS analysis showing the average hydrodynamic diameter of MXene and MXene-APTES-DFO nanosheets (b) Zeta Potential of the MAX phase, MXene, MXene-APTES, and MXene-APTES-DFO and (c) *In vitro* cytotoxicity analysis of MXene-APTES-DFO in cell lines.

**Figure 4:**
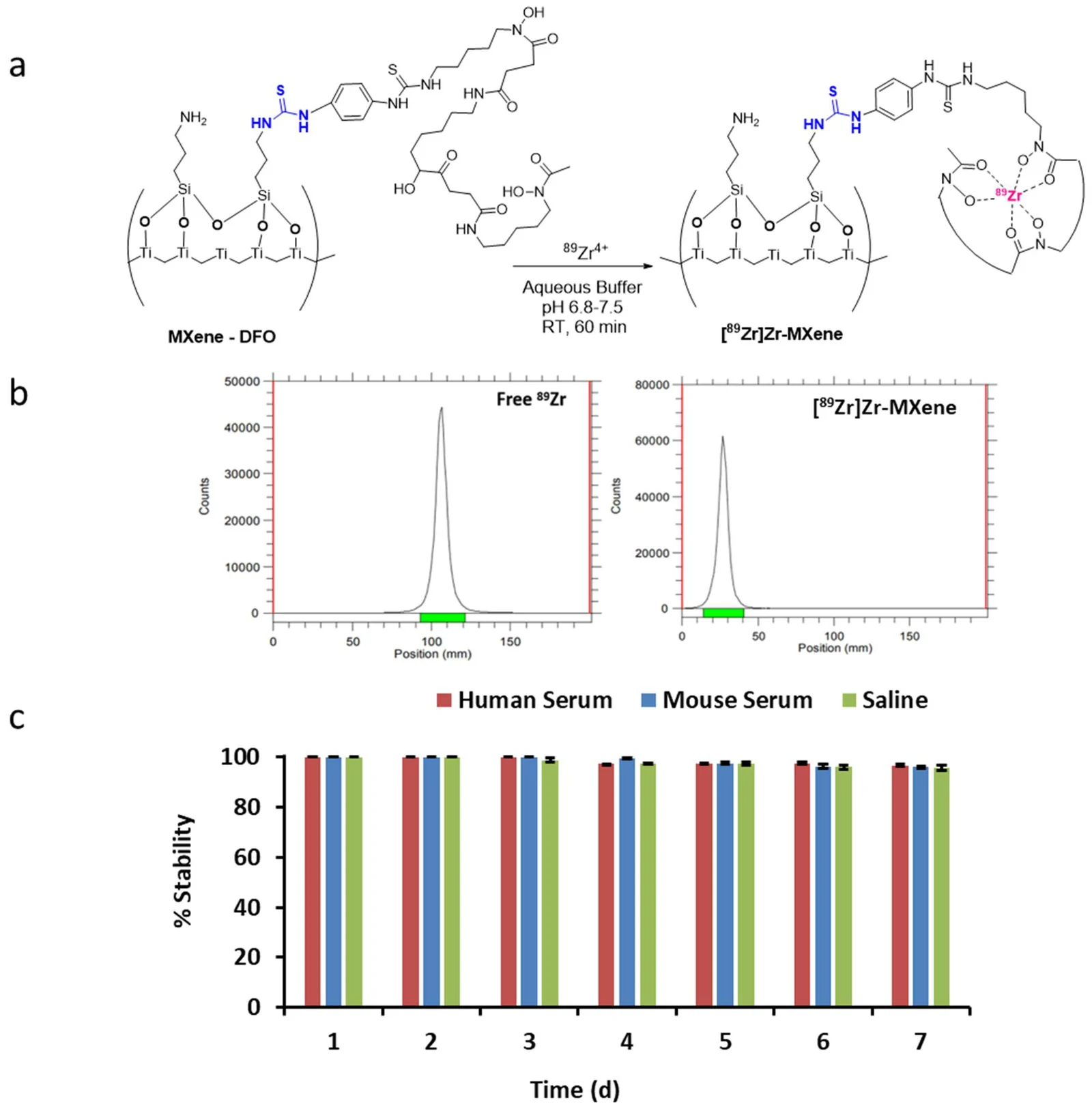
(a, b) Radiosynthesis of [^89^Zr]Zr-MXene along with instant thin-layer chromatography (iTLC) analysis of free ^89^Zr and [^89^Zr]Zr-MXene using DTPA as a mobile phase solvent; and (c) *In vitro* serum or saline stability of [^89^Zr]Zr-MXene. The combination of high radiochemical purity and excellent *in vitro* stability of [^89^Zr]Zr-MXene provided a strong foundation for subsequent *in vivo* evaluation.

**Figure 5:**
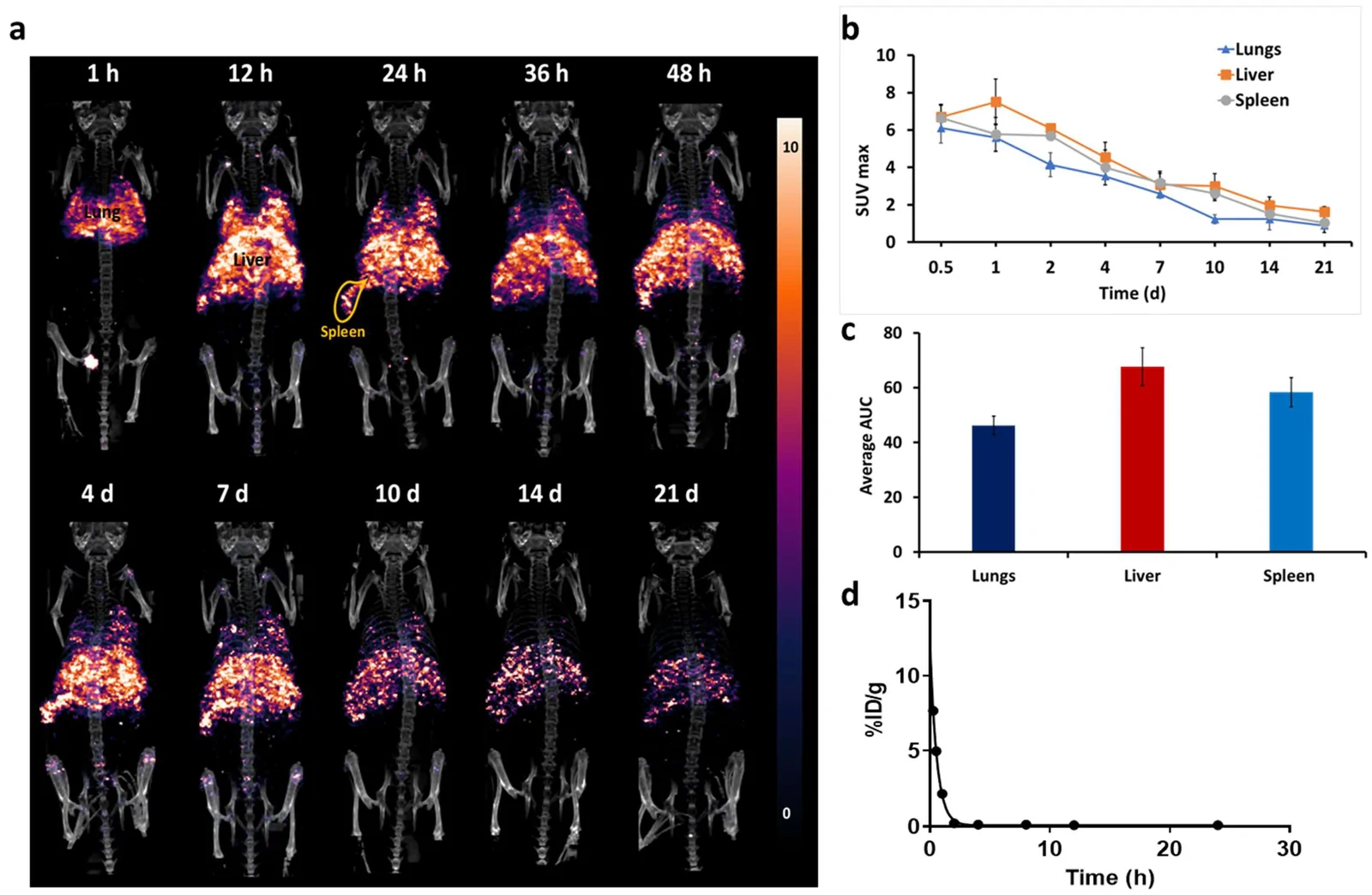
(a) *In vivo* PET/CT images of mice (n = 4) following intravenous injection of [^89^Zr]Zr-MXene (0.814 MBq); (b) quantitative PET imaging analysis (ROIs) over the lungs, liver, and spleen on reconstructed PET images at each timepoint (0.5, 1, 2, 4, 7, 10, 14, and 21 days post-injection); (c) noncompartmental pharmacokinetic analysis performed on the organ ROI data; (d) blood pharmacokinetics [^89^Zr]Zr-MXene post intravenous injection

**Figure 6:**
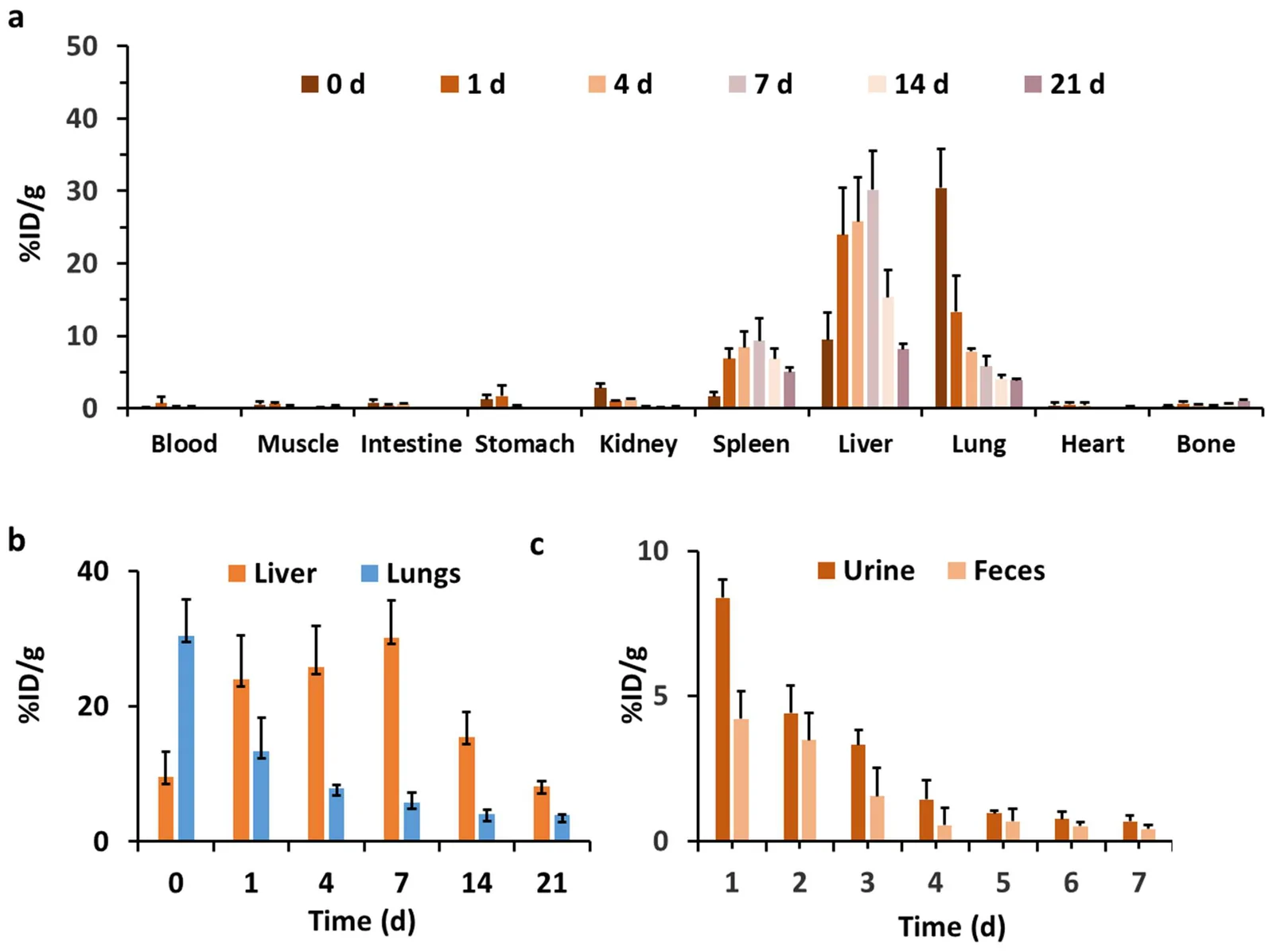
(a) Biodistribution data of [^89^Zr]Zr-MXene in BALB/c mice following intravenous injection of [^89^Zr]Zr-MXene (n = 4). Data were acquired at 0, 1, 4, 7, 14, and 21 days post-injection; (b) Organ clearance/retention kinetics in liver and lungs; and (c) [^89^Zr]Zr-MXene levels in the urine and feces during the first week post-injection. Mouse excretions were collected using metabolic cages. The error bars represent the standard deviation of 4 mice per group.

**Figure 7:**
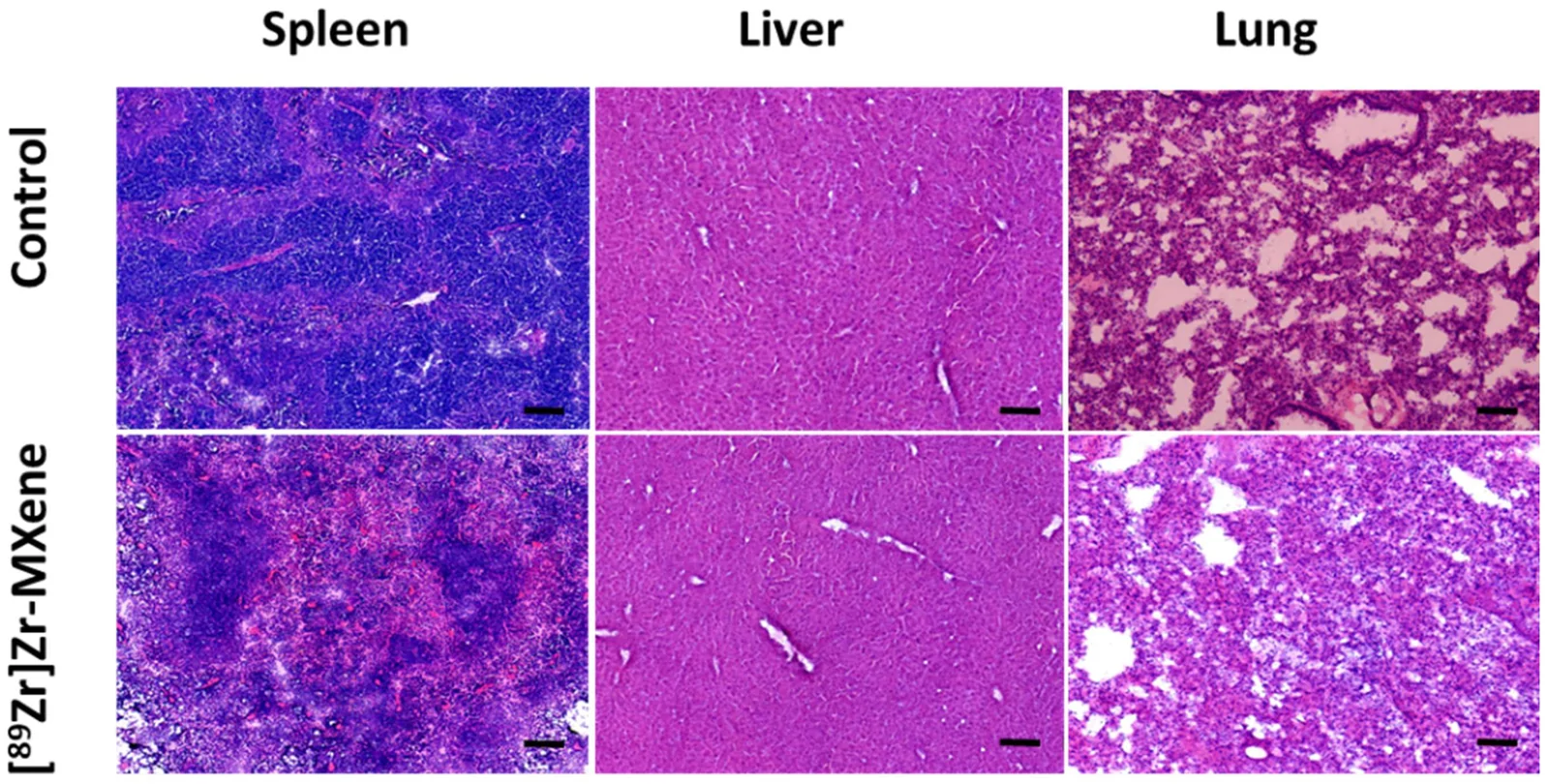
*In vivo* toxicology analysis of the [^89^Zr]Zr-MXene nanosheets. Hematoxylin and eosin staining of control and [^89^Zr]Zr-MXene nanosheets treated group. All sections were stained with H&E stain and observed under a light microscope at 40X magnification. Scale bar: 100 µm.
