# Supplementary material for: Longitudinal PET Imaging Reveals the Biological Fate, Pharmacokinetics, and Clearance of Zirconium-89-Labeled MXene Nanosheets In Vivo
Source: Nanotheranostics. 2026 Oct 9;10:269–84. doi: 10.7150/ntno.138975 (PMC13686794; doi:10.7150/ntno.138975)
Supplement: Supplementary file 1 [file ntnov10p0269s1.pdf]

Longitudinal PET Imaging Reveals the Biological Fate, Pharmacokinetics, and Clearance of Zirconium-89-Labeled MXene Nanosheets *In Vivo*

Iqra Bibi[1, 2, #], Sairan Eom[1, #], Sang Jin Han[1], Kyung Jun Kang[1], Jung Young Kim[1, 2], Sajid Mushtaq[1, 3*],

Ji-Ae Park[1, 2*]

[1]*Division of Applied RI, Korea Institute of Radiological & Medical Sciences (KIRAMS), 75 Nowon-ro, Nowon-gu, Seoul 01812, Republic of Korea*

[2]*University of Science and Technology (UST), 217, Gajeong-ro, Yuseong-gu, Daejeon 34113, Republic of Korea*

[3]*Department of Chemistry, Pakistan Institute of Engineering and Applied Sciences (PIEAS), P. O. Nilore, Islamabad 45650, Pakistan*

***Address correspondence to**

sajidmushtaq@pieas.edu.pk

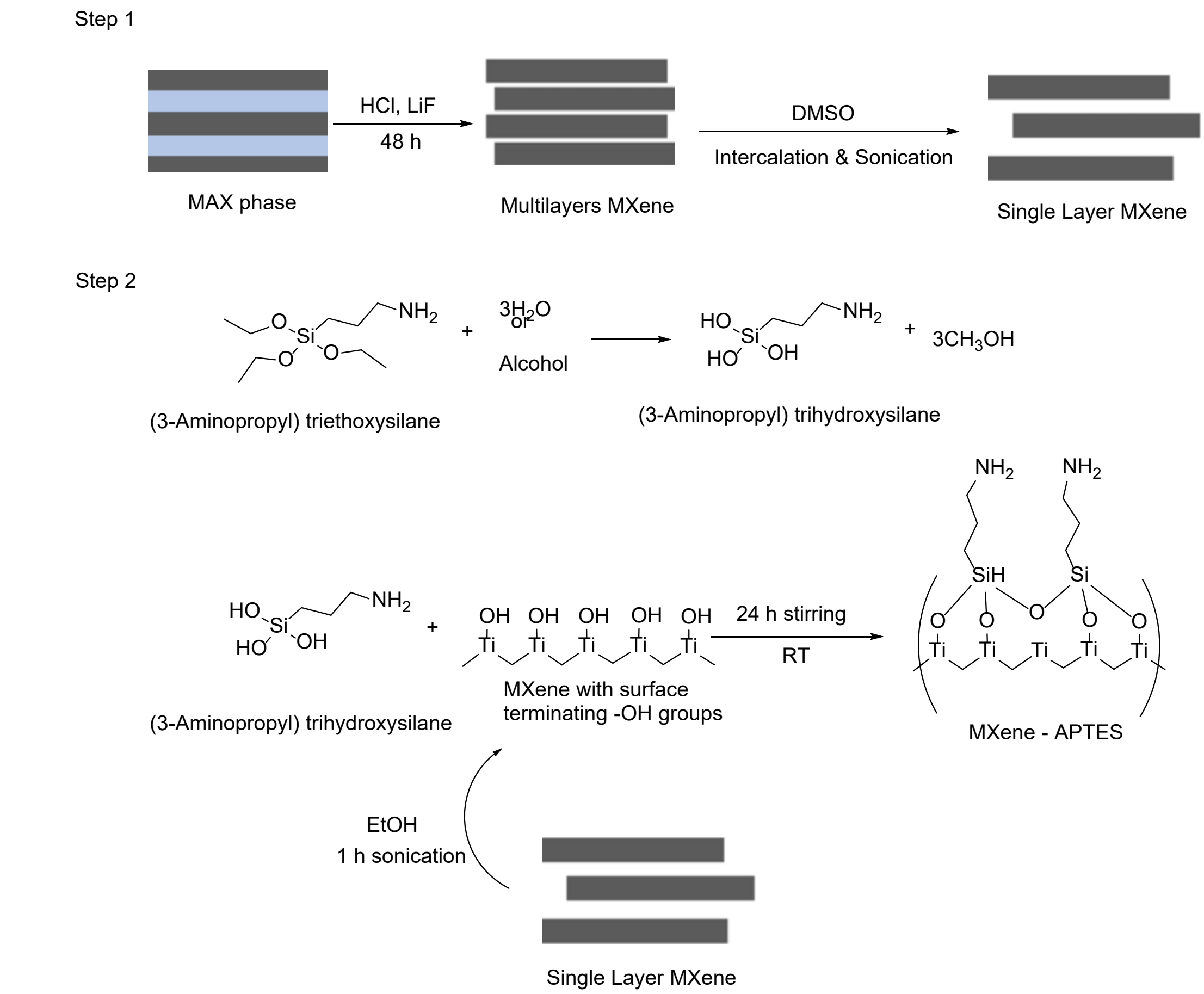

Scheme S1. Synthesis of MXene ($Ti_3C_2T_x$) and surface modification with (3-aminopropyl)triethoxysilane (APTES).

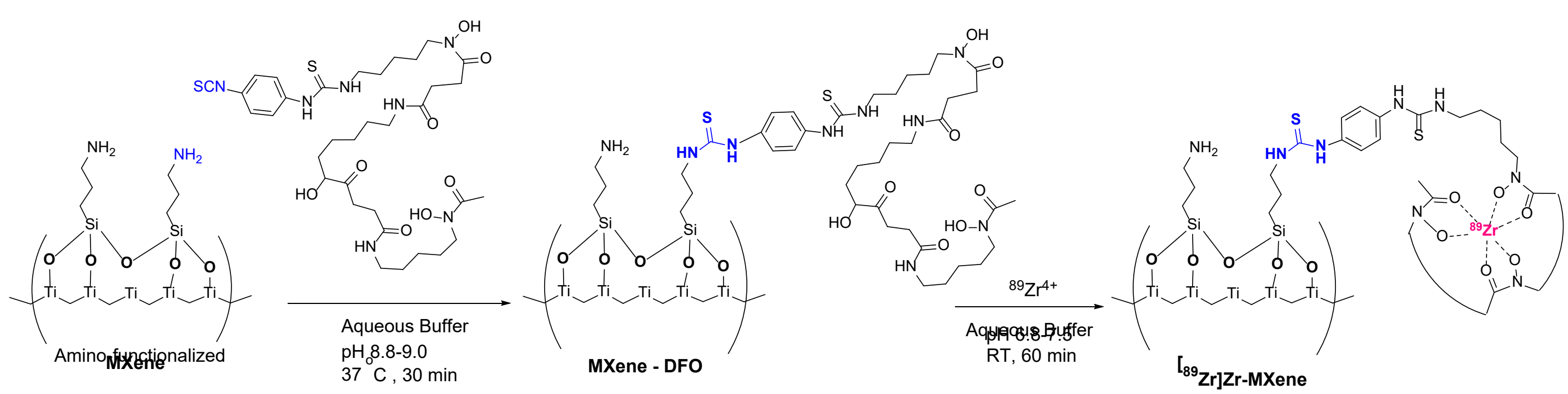

Scheme S2. Synthesis of MXene-APTES-DFO followed by its radiolabeling with ^{89}Zr to yield [^{89}Zr]Zr-MXene.

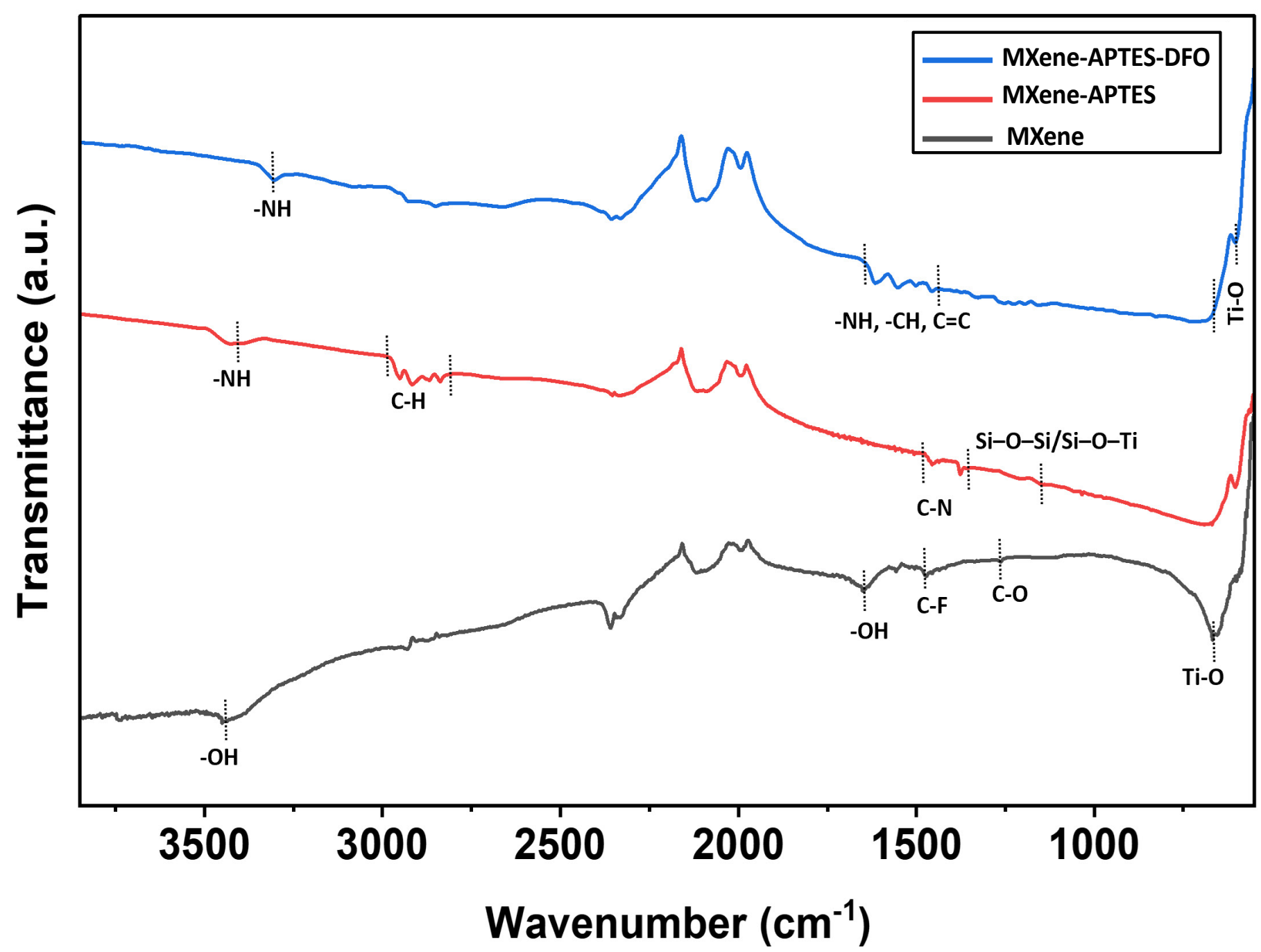

Figure S1. Fourier-transform infrared (FTIR) analysis of the MXene, MXene functionalized with (3-aminopropyl)triethoxysilane (MXene-APTES), and MXene-APTES conjugated with *p*-isothiocyanatobenzyl-desferrioxamine (MXene-APTES-DFO).

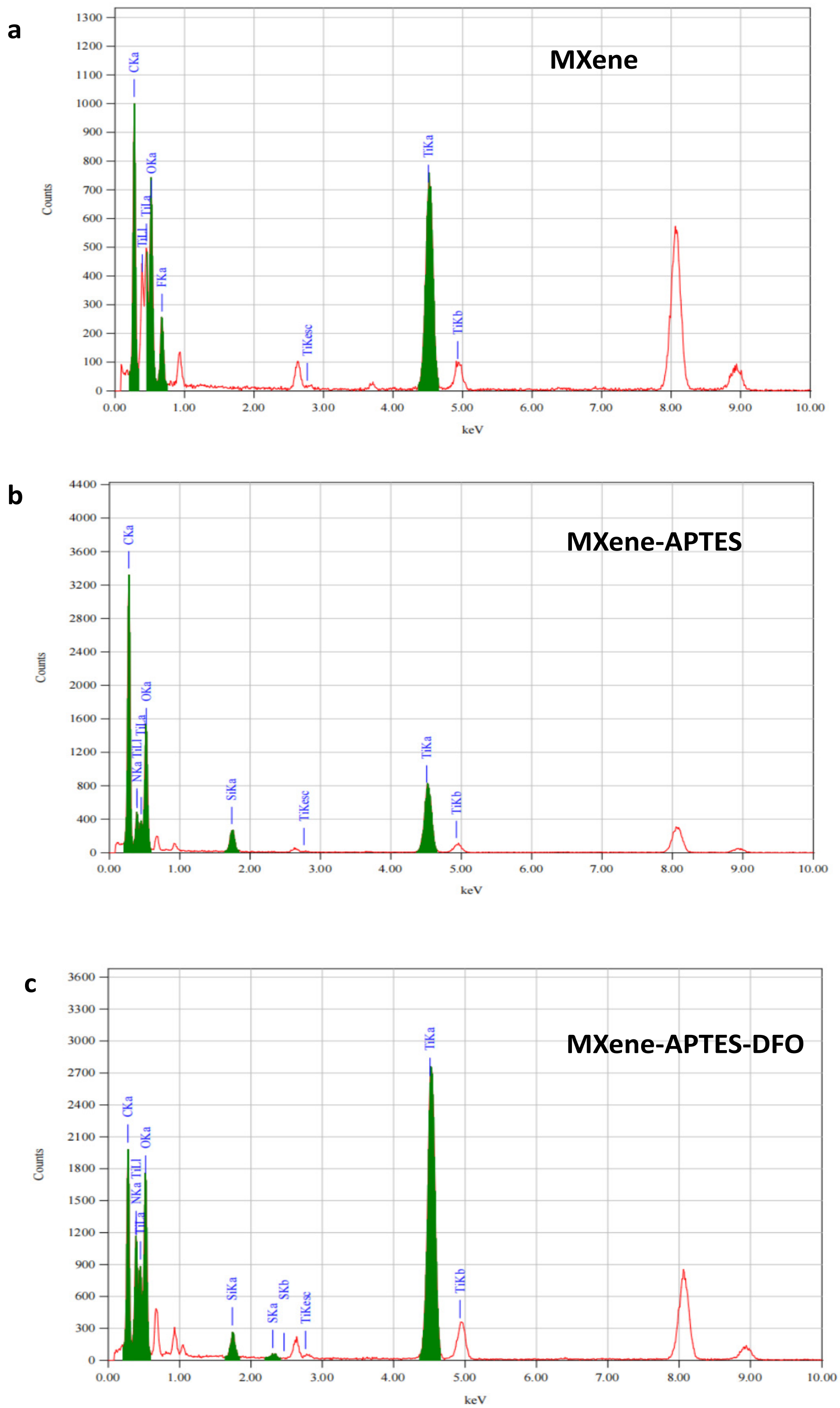

Figure S2. EDX spectra of (a) MXene, (b) MXene-APTES, and (c) MXene-APTES-DFO.

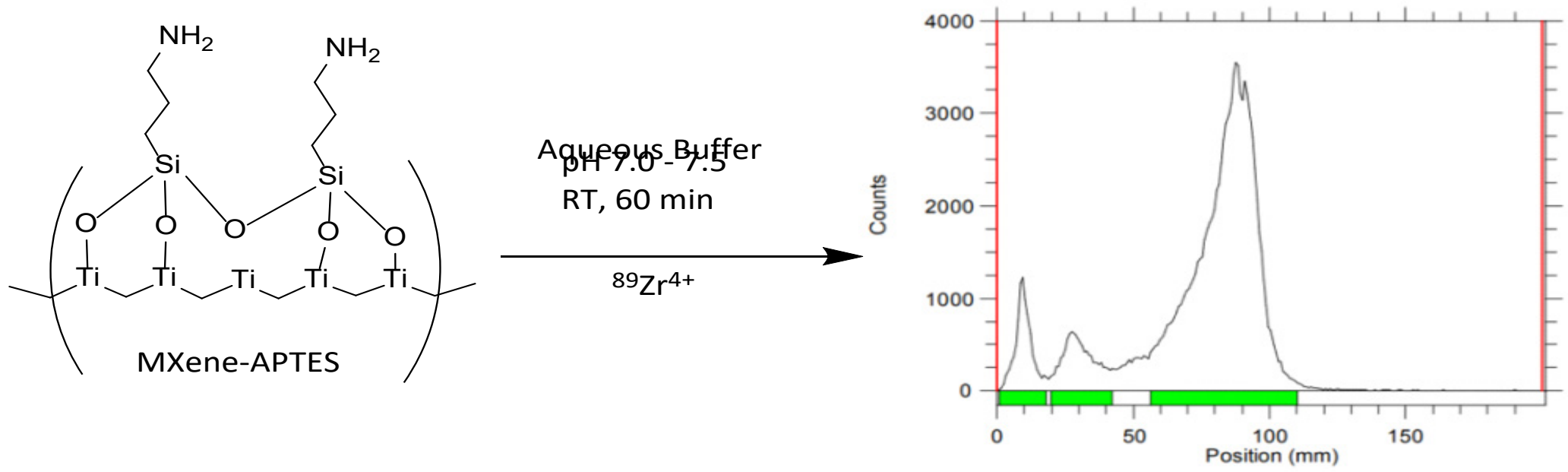

Figure S3. Radiolabeling of MXene-APTES with ^{89}Zr in the absence of DFO chelator and its characterization by iTLC. It can be seen that there is no non specific radiolabeling of MXene in the absence of DFO chelator.

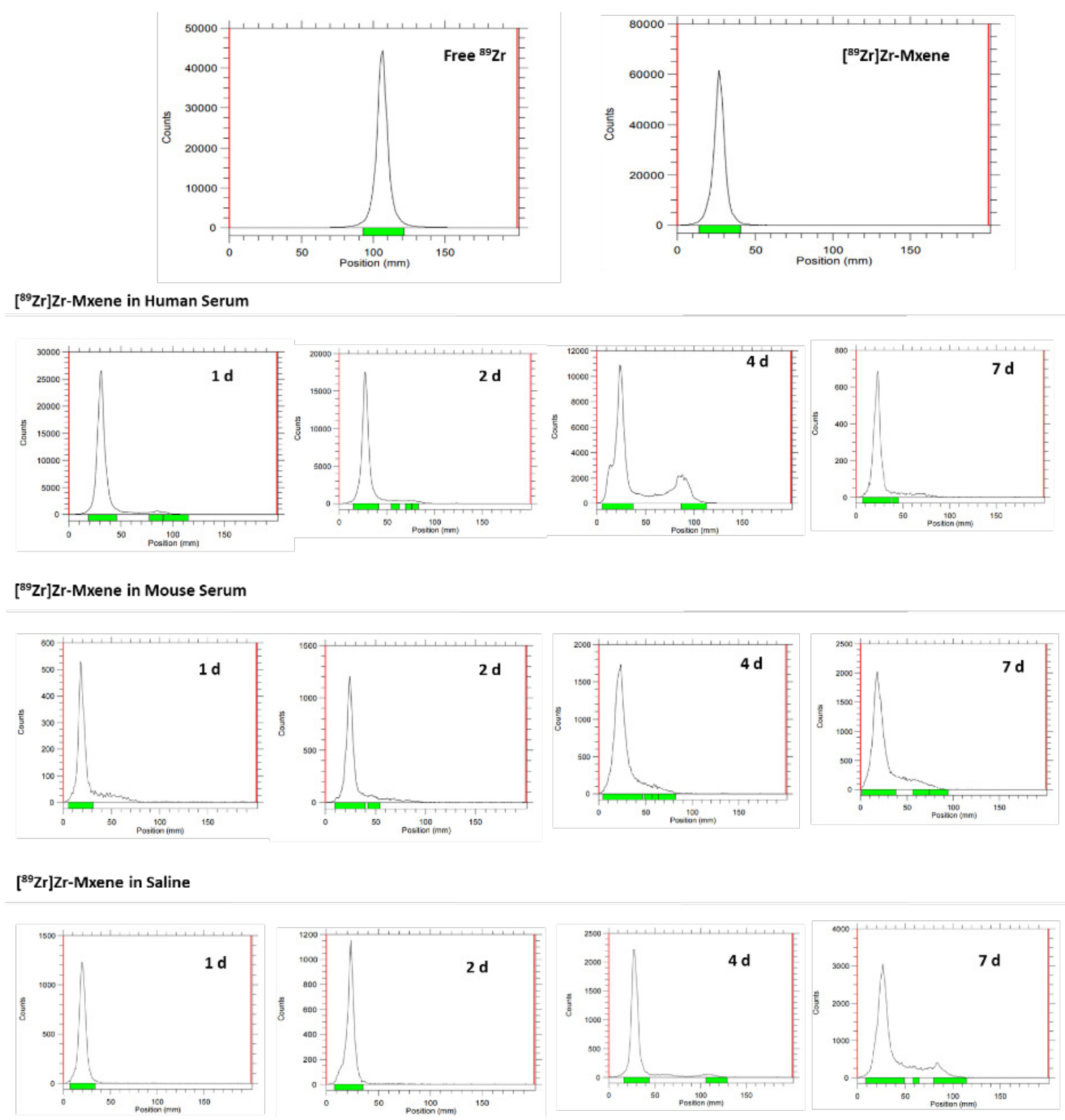

Figure S4. In vitro stability of [^{89}Zr]Zr-MXene in human serum, mouse serum, and 0.9% saline over 7 days, monitored by instant thin-layer chromatography (iTLC) using DTPA as the mobile phase. The chromatograms show minimal release of ^{89}Zr from [^{89}Zr]Zr-MXene over the entire period, indicating excellent stability of the radiolabeled complex in biological and physiological media.

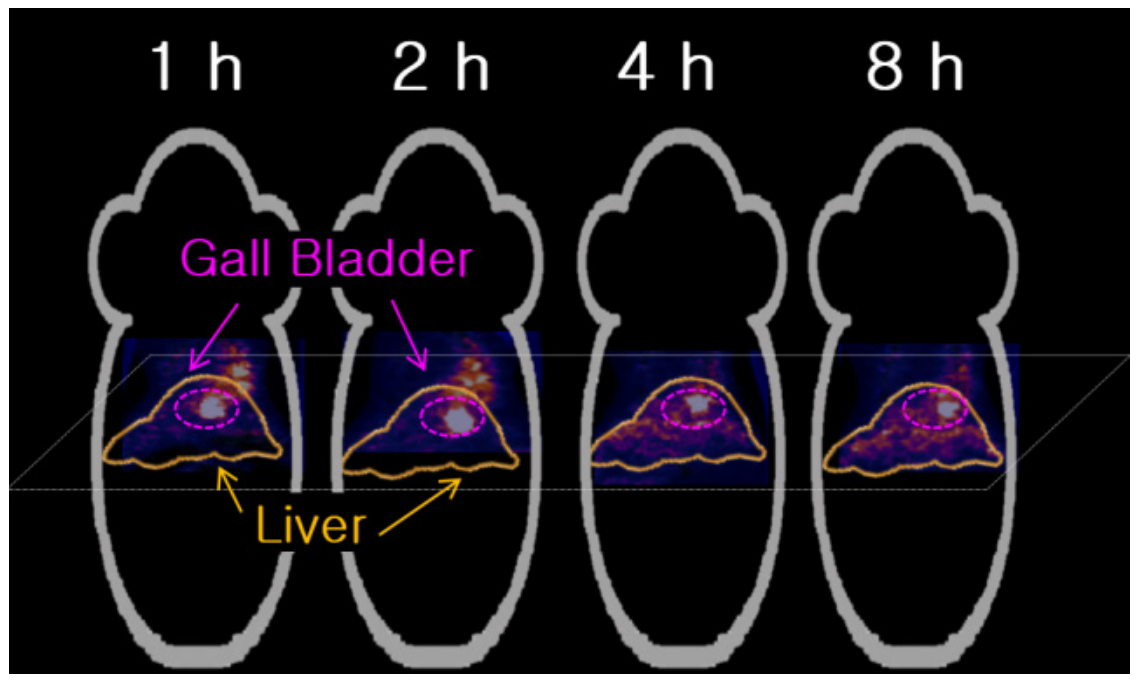

Figure S5. In vivo PET imaging showing [^{89}Zr]Zr-MXene intense activity signal in the gall bladder at the early time points (1 h to 8 h), which gradually decreased over time, while liver retention remained consistent.

Table S1: Elemental composition of MAX, MXene, MXene-APTES, and MXene-APTES-DFO determined by XPS analysis.

Element	MAX (Atomic %)	MXene (Atomic %)	MXene-APTES (Atomic %)	MXene-APTES-DFO (Atomic %)
Al	16.07	—	—	—
C	26.49	68.81	40.80	49.71
N	1.14	—	2.62	7.99
O	46.33	15.16	15.59	20.84
Ti	9.98	7.78	12.37	12.14
F	—	8.26	24.79	6.00
Si	—	—	3.29	0.91
S	—	—	—	2.40

XPS-derived surface elemental composition of MAX, MXene, MXene-APTES, and MXene-APTES-DFO. A clear shift in elemental ratios, particularly the appearance of Si and N signals and the reduction in F content after APTES and DFO functionalization, confirms successful surface modification and complexation on MXene nanosheets.

Table S2: XPS analysis results showing the elemental composition and corresponding binding energies of MAX, MXene, MXene-APTES, and MXene-APTES-DFO.

Element / Signal	MAX		MXene		MXene-APTES		MXene-APTES-DFO	
	Atomic (%)	Binding Energy (eV)	Atomic (%)	Binding Energy (eV)	Atomic (%)	Binding Energy (eV)	Atomic (%)	Binding Energy (eV)
O 1s	32.05	530.05	10.53	530.76	7.53	531.00	13.28	531.13
C 1s	10.03	284.05	44.63	284.24	13.49	284.32	25.49	284.93
Ti 2s	3.12	562.72	2.70	563.02	3.87	562.51	5.15	562.48
Ti $2p_{1/2}$	0.42	463.16	0.37	463.99	—	—	—	—
Ti $2p_{3/2}$	1.40	456.85	0.89	455.41	2.39	455.43	3.77	455.47

Ti 3s	8.47	58.80	9.83	60.89	8.75	60.24	15.72	60.59
Ti 3p	7.55	34.61	5.85	35.68	9.05	35.09	9.29	35.18
Al 2s	12.59	117.83	—	—	—	—	—	—
Al 2p	12.57	73.04	—	—	—	—	—	—
F 1s	—	—	7.07	684.22	11.84	688.11	3.83	685.44
N 1s	—	—	—	—	1.36	400.81	5.23	399.28
Si 2s	—	—	—	—	1.92	153.16	1.21	161.09
Si 2p	—	—	—	—	1.82	102.20	0.65	101.95
Na 1s	—	—	—	—	—	—	3.24	1071.29
S 2p	—	—	—	—	—	—	0.68	161.45

XPS elemental composition and binding energy distribution of surface species in MAX, MXene, MXene-APTES, and MXene-APTES-DFO 2D nanosheets. The appearance of N 1s, Si 2p/2s, and S 2p peaks confirms successful surface modification and coordination through APTES and *p*-SCN-Bz-DFO functionalization.

Table S3: Zeta potential (mV) values of MAX, MXene, MXene-APTES, and MXene-APTES-DFO. The alternating positive and negative surface charges reflect successive surface modifications from Al etching, silane functionalization, and DFO conjugation.

**Sample**	**Zeta Potential (mV) ± SD**
MAX	33.48 ± 6.36
MXene	−24.08 ± 1.65
MXene-APTES	14.32 ± 2.63
MXene-APTES-DFO	−28.93 ± 1.94

Table S4: Biodistribution of [^{89}Zr]Zr-MXene in mice at various time points post i.v. administration. Values represent %ID/g ± SD (n = 4).

	**0 d**	**1 d**	**4 d**	**7 d**	**14 d**	**21 d**
**Blood**	0.11 ± 0.08	0.74 ± 0.88	0.12 ± 0.117	0.22 ± 0.09	0.02 ± 0.003	0.04 ± 0.01
**Muscle**	0.53 ± 0.40	0.57 ± 0.18	0.25± 0.14	0.04 ± 0.007	0.08 ± 0.02	0.30 ± 0.13
**Intestine**	0.77 ± 0.38	0.39 ± 0.20	0.59 ± 0.13	0.02 ± 0.01	0.03 ± 0.01	0.02 ± 0.007
**Stomach**	1.21 ± 0.62	1.68 ± 1.43	0.31 ± 0.17	0.04 ± 0.03	0.04 ± 0.006	0.06 ± 0.01
**Kidney**	2.82 ± 0.59	1.00 ± 0.08	1.18 ± 0.15	0.23 ± 0.04	0.10 ± 0.02	0.16 ± 0.06
**Spleen**	1.61 ± 0.61	6.82 ± 1.37	8.41 ± 2.24	9.31 ± 3.17	6.86 ± 1.38	4.99 ± 0.62
**Liver**	9.48 ± 3.77	23.92 ± 6.49	25.75 ± 6.11	30.16 ± 5.42	15.36 ± 3.71	8.13 ± 0.72
**Lung**	30.43 ± 5.39	13.30 ± 4.96	7.81 ± 0.49	5.78 ± 1.47	4.01 ± 0.62	3.86 ± 0.19
**Heart**	0.36 ± 0.46	0.50 ± 0.35	0.41 ± 0.34	0.05 ± 0.02	0.06 ± 0.02	0.23 ± 0.02
**Bone**	0.29 ± 0.06	0.65 ± 0.23	0.41 ± 0.18	0.38 ± 0.06	0.49 ± 0.24	1.04 ± 0.14

Table S5: DLS distributions of MXene-APTMS-DFO after 24 h in various media

**Medium**	**Hydrodynamic diameter (nm)**	**PDI**
Water	300 ± 55	0.21
0.9% saline	297 ± 48	0.22
90% mouse serum	312 ± 51	0.22

Table S6: Injection parameters for the *in vivo* PET imaging and biodistribution studies**.**

**Sr.No.**	**Parameter**	**Values**
1	Injected radioactivity	0.185 MBq/mouse
2	Injected MXene-APTES-DFO	5 µg/mouse
3	Average mouse body weight	22 g
4	Administered dose	0.23 mg/Kg
5	Radiochemical yield	>95%
6	Radiochemical purity	99.39 ± 0.66% (n = 5)
7	Final specific activity	141 MBq/mg
8	Injection vehicle	0.9% Saline
